# Mechanism of Alternating Poly(lactic-*co*-glycolic acid) Formation by Polymerization of (*S*)- and (*R*)-3-Methyl Glycolide Using an Enantiopure Aluminum Complex

**DOI:** 10.1021/acscatal.3c04955

**Published:** 2023-12-18

**Authors:** Yolanda Rusconi, Massimo Christian D’Alterio, Claudio De Rosa, Yiye Lu, Sarah M. Severson, Geoffrey W. Coates, Giovanni Talarico

**Affiliations:** † Scuola Superiore Meridionale, Largo San Marcellino, 80138 Napoli, Italy; ‡ Dipartimento di Scienze Chimiche, Università degli Studi di Napoli Federico II, 80126 Napoli, Italy; § Department of Chemistry and Chemical Biology, Baker Laboratory, Cornell University, Ithaca, New York 14853-1301, United States

**Keywords:** Ring-opening polymerization, sequence-control, regioselective MeG polymerization, DFT calculations, enantiopure catalyst

## Abstract

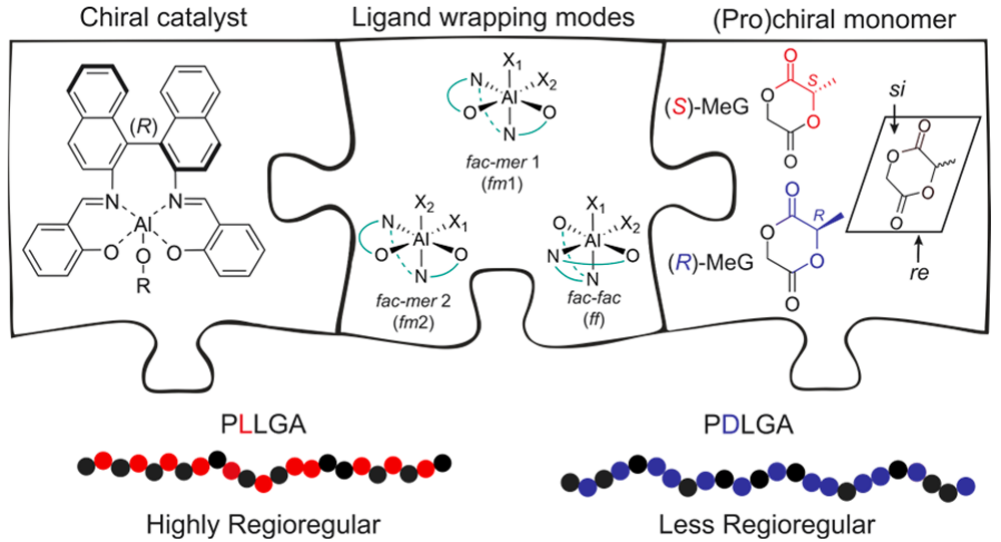

The mechanism(s) of alternating PLGA synthesis by ring-opening polymerization of (*S*)- and (*R*)-3-methyl glycolide promoted by enantiopure aluminum complexes have been rationalized by density functional theory (DFT) calculations. The high regioselectivity of the (*S*)-MeG polymerization is obtained by repetitive ring opening at the glycolyl site by the (*R*)-catalyst whereas a lower regioselectivity is predicted by the ROP of (*R*)-MeG. The behavior of the two monomers is rationalized by unveiling the active site fluxionality of the enantiopure catalyst, identifying the rate-limiting steps that encode a preference at the glycolyl site versus the lactyl site, and revealing selection of the opposite monomer enantioface. The microstructure of the PLGA copolymers is predicted by considering the influence of the configuration of the last inserted unit. The identification of the preferred mechanistic paths may allow for a targeted catalyst design to enhance control of the polymer microstructures.

Biocompatible materials such as poly(glycolic acid)^1^ (PGA), poly(lactide)^2^ (PLA) and their copolymers, poly(lactic-*co*-glycolic acid)^3^ (PLGA), are widening their range of application in the biomedical field. In particular, the degradation of PLGA via hydrolysis and consequent participation of the metabolite monomers in the Krebs cycle of the human body^4^ allows the use of this polymer for tissue engineering^5^ and drug delivery employments,^6^ as confirmed by the Food and Drug Administration (FDA) approval.^7^ The ring-opening polymerization (ROP) of the cyclic cross-dimer of glycolic and lactic acid, 3-methyl glycolide (3-MeG), has been identified as the most powerful method to synthesize alternating PLGA.^8,9^ Indeed, this monomer displays two possible attack sites: sites A (lactyl site) and B (glycolyl site) (Figure 1). The repetitive ring opening at only one site translates to high sequence control, a key factor that directly influences the degradation rate of the polymer and enables formation of a perfectly alternating copolymer^10^ with controllable molecular weights.^11^ Regioselective catalysts employed in the past years include stannous octanoate (Sn(Oct)_2_)^12^ and organocatalytic systems (phosphazene base, P_2_-*t*-Bu).^13^ Very recently, alternating PLGA with high regioselectivity has been obtained by Coates and Meyer^14,15^ through ROP of (*S*)-MeG promoted by (*R*)-(SalBinap)AlOR ((*R*)-**2**, Figure 1) by a site-controlled coordination–insertion mechanism with nearly exclusive ring opening at the glycolyl acyl–O bond site. Interestingly, a lower regioselectivity was obtained when (*S*)-**2** was used in combination with (*S*)-MeG (corresponding to (*R*)-**2** and (*R*)-MeG in Figure 1) due to a catalyst mismatch. Previously, we used DFT to study the origin of stereocontrol of (*R*)-**2** in the isoselective ROP of *rac*-LA^16−18^ and in the syndioselective ROP of *meso*-LA^19^ (Figures 1a and 1b).^20,21^ Intrigued by the high regioselectivity maintained by (*R*)-**2** in the ROP of MeG, we have performed an in-depth computational analysis to further inform the origin of regiocontrol and rationalize the ROP mechanism.

**Figure 1:**
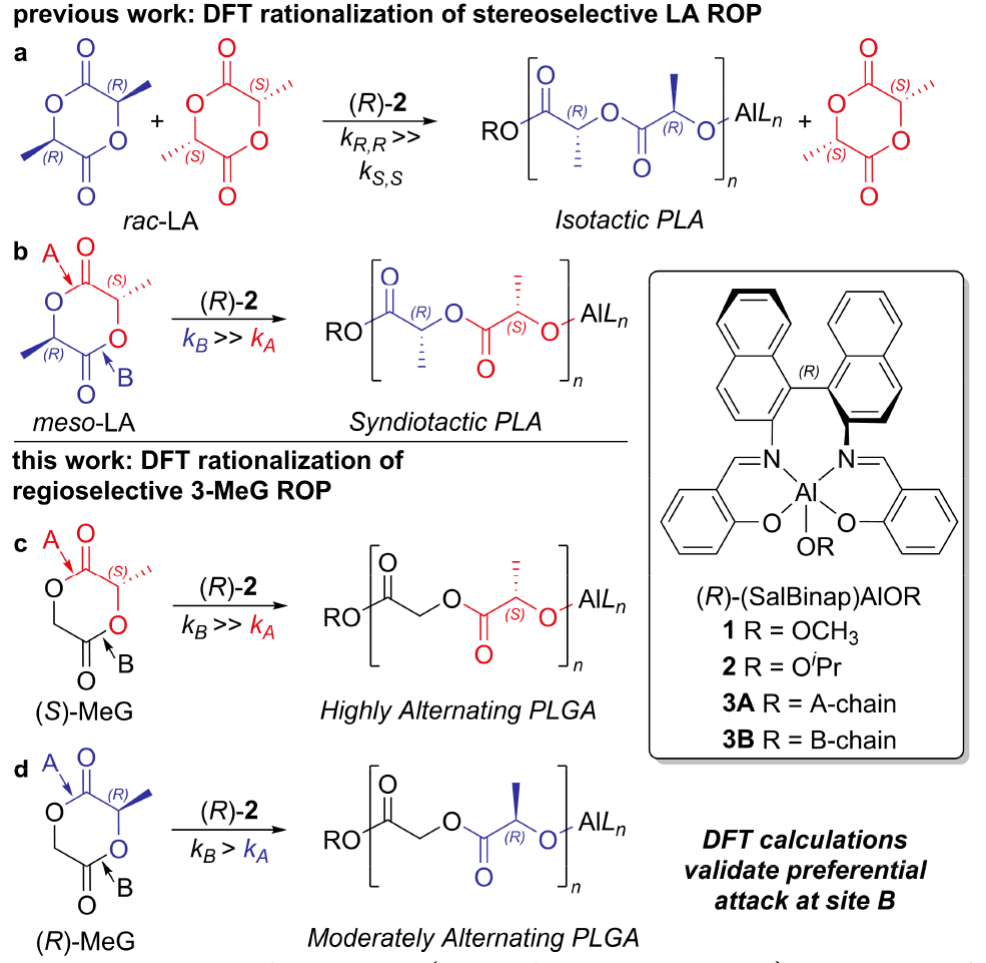
(a, b) Stereoselective LA (previous work) and (c, d) regioselective 3-MeG (this work) ROP by (*R*)-(SalBinap)AlOR. (*S*)-LA, (*R*)-LA and GA are colored red, blue, and black, respectively.

The simplified ROP MeG mechanism at the two attack sites is summarized in Scheme 1, with two transition states (TSs) arising from the nucleophilic addition of the OR at the carbonyl carbon (**TS1**), followed by the ring opening of the monomer via the cleavage of the bond between the carbonyl carbon and the endocyclic oxygen in the α-position (**TS2**, Scheme 1). It is worth recalling that the (*R*)-**2** TS compounds may show two different wrapping modes: *fac-fac* (*ff*) and *fac-mer* (*fm*). The latter can have two conformations, namely, *fm*1 and *fm*2, characterized by the position of the polymer chain (OR) *trans* to the N and O atoms, respectively (Scheme 2).

**Scheme 1:**
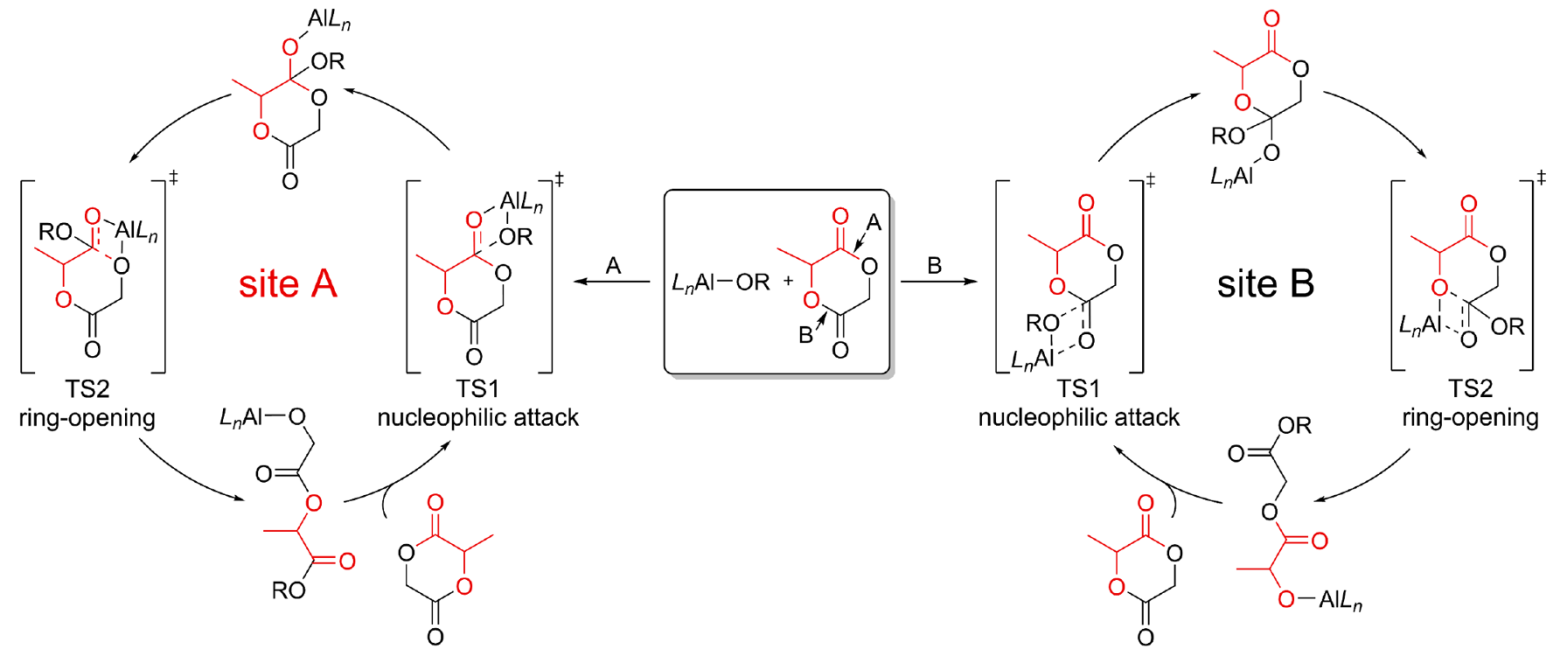
Catalytic Cycle for 3-MeG ROP at Site A (Left) and Site B (Right) The lactyl and glycolyl sites are colored red and black, respectively.

**Scheme 2:**
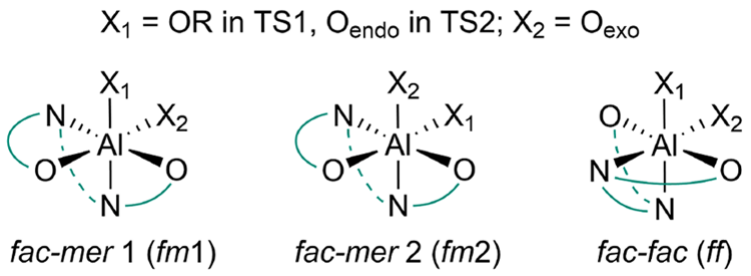
Schematic View of the TSs Catalyst Configurations O_endo_ and O_exo_ refer to the endocyclic O atom of the monomer and the carbonyl O atom, respectively.

Furthermore, the two (*R*)-MeG and (*S*)-MeG enantiomers may have the methyl substituent occupying the axial or equatorial position (Figure S1 in the Supporting Information) and each of these enantiomers displays two enantiofaces (*re* and *si*). Therefore, eight different possible attacks at each (*R*)-MeG and (*S*)-MeG have been considered in both TS1 and TS2. The combination of all elements of chirality to analyze the regioselective ROP mechanisms of MeG is reported in Figure S2 in the Supporting Information. We investigated all possible reaction paths, as summarized here: a preservation of catalyst configuration throughout the catalytic cycle (**M1**, with three different paths designated with the capital letters A, B, and C; see Figure S3 in the Supporting Information); an exchange between monomer and growing chain positions moving from TS1 to TS2 (**M2**) (2 paths, Figure S4 in the Supporting Information); and a change in the wrapping mode of the ligand around the active site during the reaction path (**M3**) (4 paths and the identification of a new TS structure, TSα; see Figure S5 in the Supporting Information). The complete study of this reaction, indeed, involves a huge number of TSs and minima structures, and in this communication, we are summarizing the main results. The DFT computational approach (geometry optimizations at B3LYP/6-311G(d,p)/SVP level of theory and single-point calculations with (B3LYP-D3BJ(CPCM)/6-311G(d,p)) has already been tested in stereoselective ROP^20,21^ and olefin polymerization.^22,23^ Details are reported in the Supporting Information, including the variability of the results depending on the functional used. The minimum energy paths (MEPs) for (*S*)-MeG insertion into (*R*)-**1** are reported in Table 1, specifying the attack site, reaction paths (first column), wrapping modes of the TSs with the preferred monomer enantioface (column 2), and the Gibbs energies of the TSs referred to (*R*)-**1** + monomer (columns 3–5). We summarize the preferred paths for mechanisms **M1**–**M3** in Table 1, whereas the complete lists are reported in Tables S1 and S2 in the Supporting Information. These values are calculated without considering the chirality of the growing chains and furnish a first estimate of the effects leading to chirality-directed regioselectivity.

**Table 1:** DFT Gibbs Energies (Δ*G*, in kcal·mol^–1^) of the MEPs for (*S*)-MeG Insertion promoted by (*R*)-1. Preferred Paths are Reported in Bold

		DFT Gibbs Energy, Δ*G* (kcal mol^–1^)		
path	wrapping mode, TS1–TS2	TS1	TSα	TS2
**Site A**				
**M1-C**	***ff–ff*(*si*)**	**10.0**		**10.1**
M2-A	*fm*1–*fm*2 (*re*)	10.7		5.0
M3-B	*fm*2–*ff* (*si*)	6.6	13.0	10.1
**Site B**				
M1-B	*fm*2–*fm*2 (*si*)	7.1		8.9
**M2-A**	***fm*1–*fm*2 (*re*)**	**7.5**		**6.3**
M3-B	*fm*2–*ff* (*si*)	7.1	11.9	10.0

The rate-limiting step (RLS) for (*S*)-MeG ROP is nucleophilic addition (TS1; see Table 1) at site B, following a **M2-A** path that connects **TS1** (*fm*1, 7.5 kcal mol^–1^) to **TS2** (*fm*2, 6.3 kcal mol^–1^). The initiation reaction on site A is limited by the high-lying **M1-C** path that preserves the *ff* conformation and displays the ring opening as the RLS (10.1 kcal mol^–1^).

The regioselectivity is predicted as the ΔΔ*G* difference between the RLS activation energies of the two possible sites, so we concluded that (*R*)-**1** is regioselective toward the first insertion of (*S*)-MeG, with a preference for site B of 2.6 kcal mol^–1^ (see Figure S6 and Tables S1–S3 in the Supporting Information).

The greater stability of TS1 at site B is due to the absence of ligand–monomer repulsion, which is the main cause of the increase in the energy of the site A species. Indeed, a remarkable steric hindrance arises from the proximity of the methyl substituent of (*S*)-MeG to the ligand when site A is attacked (Figure S7 in the Supporting Information).

The analogous results for the first (*R*)-MeG insertion promoted by (*R*)-**1** are reported in Table 2. The MEPs calculated for the ROP of (*R*)-MeG (**M2-B** for site A and **M1-B** for site B, Figure S8 in the Supporting Information) remark on a low regioselectivity due to the small preference for site B attack (complete lists are given in Tables S4–S6 in the Supporting Information). Overall, the calculations of the first MeG insertion agree with the experimental trend reported by Coates, who tested the two enantiomers of complex **2** with the (*S*)-configured monomer and reported that (*S*)-**2** exhibited a lower regioselectivity with (*S*)-MeG compared to (*R*)-**2** due to a chirality mismatch.^14^ Furthermore, this trend is also reproduced by changing the computational protocol (Table S7 in the Supporting Information).

**Table 2:** DFT Gibbs Energies (Δ*G*) of the MEPs for (*R*)-MeG Insertion Promoted by (*R*)-1^a^

		DFT Gibbs Energy, Δ*G* (kcal mol^–1^)		
path	wrapping mode of TS1–TS2	TS1	TSα	TS2
**Site A**				
M1-A	*fm*1–*fm*1 (*re*)	6.5		9.0
**M2-B**	***fm*1–*fm*2 (*re*)**	**6.5**		**8.2**
M3-B	*fm*2–*ff* (*si*)	8.8	10.3	7.4
**Site B**				
**M1-B**	***fm*2–*fm*2 (*si*)**	**6.3**		**7.9**
M2-A	*fm*1–*fm*2 (*si*)	7.5		7.9
M3-B	*fm*2–*ff* (*re*)	7.9	11.4	7.6

a Preferred paths are reported in bold.

At this stage, note that the preferential MeG ring opening at site B has been experimentally proven by performing a [MeG]:[(*R*)-**2**] = 1:1 ring-opening experiment and investigating the ratio of the site A and B chain ends in the initial ring-opened adducts.^14^ In order to conduct a direct comparison between the calculations and the NMR regioselectivity reported (96% yield of lactyl-terminated product),^14^ we also investigated the insertion of (*S*)-MeG and (*R*)-MeG at (*R*)-**2** with O^*i*^Pr as the initiator of the reaction. The results are reported in Tables S8 and S9 in the Supporting Information and they confirm the higher regioselectivity obtained on (*S*)-MeG with respect to (*R*)-MeG. However, while the regioselective ROP of (*S*)-MeG promoted by (*R*)-**1** and (*R*)-**2** may appear straightforward (site B showing a clear preference over site A), more complicated is the ROP of (*R*)-MeG, which shows a lower regioselectivity, depending on the identity of the growing chain.

To achieve a final picture, we decided to calculate the propagation paths for (*S*) and (*R*)-MeG with both A-chain and B-chain arising, respectively, from the attack at site A and site B during the first insertion. This step is mandatory to validate the regioselectivity and to assess the role of the polymer chain configuration on the whole mechanism. The results are summarized in Tables 3 and 4 for (*S*) and (*R*)-MeG polymerization, respectively.

**Table 3:** DFT Gibbs Energies (Δ*G*) of the MEPs for (*S*)-MeG Propagation Promoted by (*R*)-3B^a^

		DFT Gibbs Energy, Δ*G* (kcal mol^–1^)		
path	wrapping mode of TS1–TS2	TS1	TSα	TS2
**Site B at B-Chain**				
M1-A	*fm*1*–fm*1 (*re*)	14.5		17.8
**M2-A**	***fm*1–*fm*2 (*si*)**	**16.3**		**15.8**
M3-A	*fm*1–*ff* (*si*)	16.3	17.5	18.2
**Site A at B-Chain**				
**M1-A**	***fm*1–*fm*1 (*si*)**	**16.6**		**18.8**
M2-B	*fm*2–*fm*1 (*si*)	20.1		18.8
M3-A	*fm*1–*ff (re)*	21.6	17.7	14.3

a Preferred paths are reported in bold.

**Table 4:** DFT Gibbs Energies (Δ*G*) of the MEPs for (*R*)-MeG Propagation Promoted by (*R*)-3B^a^

		DFT Gibbs Energy, Δ*G* (kcal mol^–1^)		
path	wrapping mode of TS1–TS2	TS1	TSα	TS2
**Site B at B-Chain**				
M1-A	*fm*1*–fm*1 (*re*)	16.4		18.2
**M2-A**	***fm*1–*fm*2 (*re*)**	**16.4**		**16.7**
M3-A	*fm*1–*ff* (*re*)	16.4	18.6	15.6
**Site A at B-Chain**				
M1-B	*fm*2–*fm*2 (*si*)	21.2		16.3
**M2-A**	***fm*1–*fm*2 (*re*)**	**15.0**		**17.9**
M3-D	*ff*–*fm*2 (*re*)	20.9	18.4	17.9

a Preferred paths are reported in bold.

Preference for site B during ROP of (*S*)-MeG is again confirmed when considering a B-chain (coming from (*S*)-MeG insertion at site B) with the mechanisms reported in Table 3 (complete lists are given in Tables S10–S14 in the Supporting Information).

They, in fact, show that the preferred mechanism is **M2-A** based on the nucleophilic attack (**TS1**, 16.3 kcal mol^–1^, Figure 2A) followed by ring opening (**TS2**, 15.8 kcal mol^–1^, Figure 2B). Recall that an analogous mechanism (*fm*1 for **TS1**, followed by a *fm*2 for **TS2**) has been suggested as the main origin of the stereoselectivity for the *rac*-LA promoted by (*R*)-SalBinam-Al system.^18^ Occasional regiodefects (preferred path of site A at the B-chain; see Table 3) are due to the **M1-A** path showing the **TS1** with similar energy to the RLS of B site (16.6 kcal mol^–1^; see Figure 2C) but with the **TS2** as the RLS (18.8 kcal mol^–1^; see Figure 2D). The latter displays several ligand–chain interactions absent or weaker in the RLS of the site B + B-chain insertion (Figure 2A–D).

**Figure 2:**
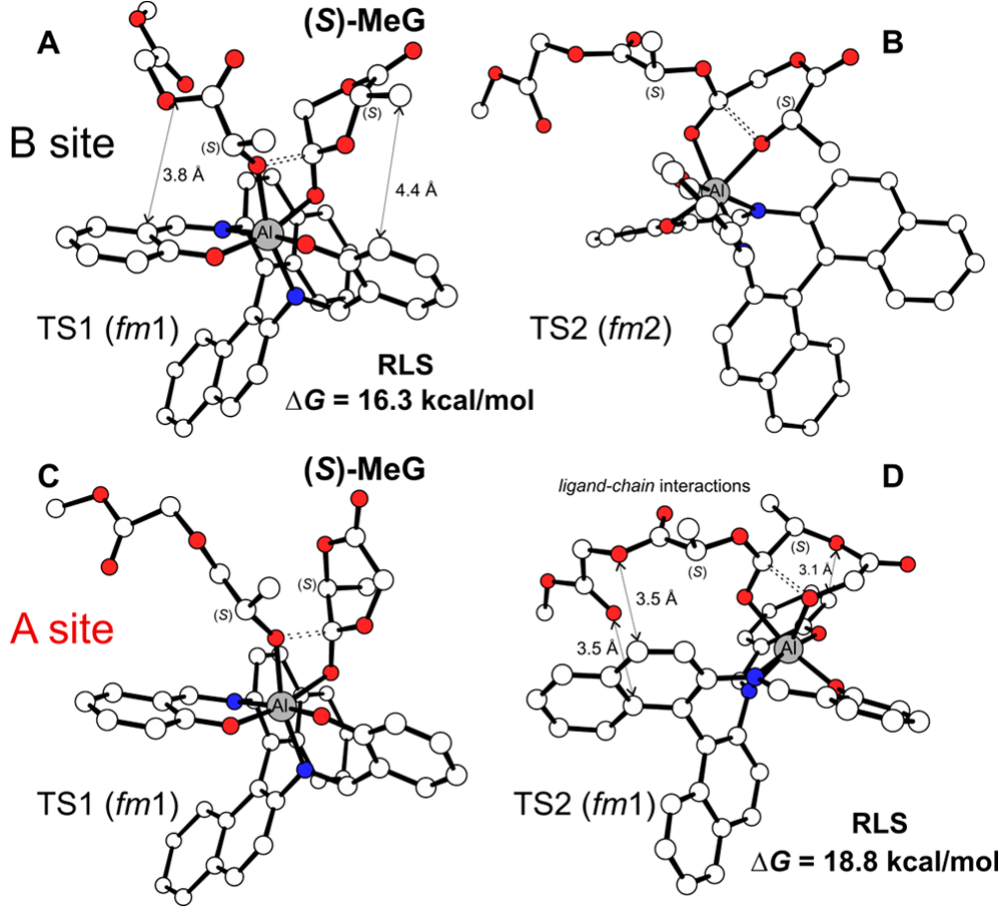
DFT geometries for the preferred path mechanisms of (*S*)-MeG insertion TSs at (A, B) site B and (C, D) site A promoted by (*R*)-**3B** system. H atoms are omitted for the sake of clarity.

The calculated regioselectivity (ΔΔ*G*_regio_ = 2.5 kcal mol^–1^) agrees with the experimental one (96%) reported in a recent paper.^14^

The results for (*R*)-MeG insertion into a B-chain reported in Table 4 (ΔΔ*G*_regio_ = 1.2 kcal mol^–1^) account for the experimental lower regioselectivity (78%)^14^ due to the close similarity of the site B (Figure 3A) and site A (Figure 3B) attacks, both showing TS2 as the RLS (see Tables S15–S20 in the Supporting Information) and with the latter less penalized than (*S*)-MeG (compare Figure 3B with Figure 2D). The prediction of the whole copolymer microstructure is achieved by considering *all* possible MeG insertions into both A- and B-chains, as specified in Table 5 (results with the B-chain are repeated for the sake of readability). Examining the insertion of (*R*)- vs (*S*)-MeG into an A-chain (arising from ring-opening of MeG at site A) also reveals differences in the resulting copolymer microstructures (Figure S9). In fact, during ROP of (*S*)-MeG, occasional regiodefects are corrected by the catalyst, as site B insertion is preferred with *both* the A- and B-chains (Table 5). For (*R*)-MeG, the regioselectivity is critically dependent on the last-inserted unit and the (lower) regioselectivity is retained with the A- and B-chains, although with opposite preference (site A attack preferred on the A-chain and site B on the B-chain; see Table 5).

**Figure 3:**
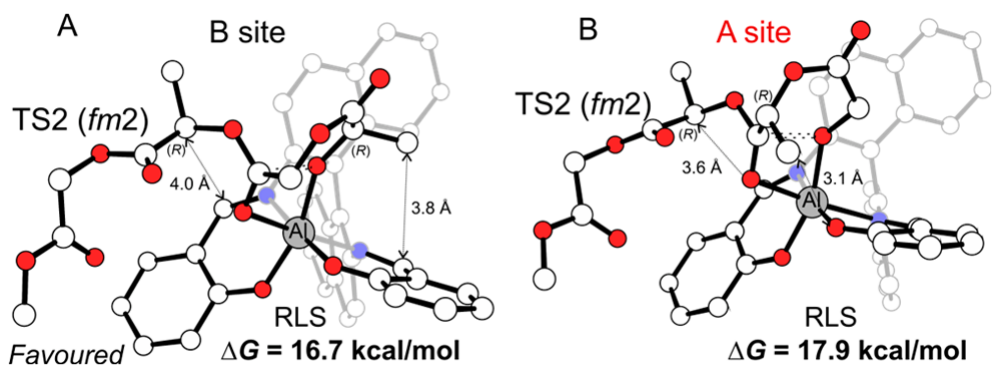
DFT geometries for the RLS of (*R*)-MeG insertion TSs at (A) site B and (B) site A promoted by the (*R*)-3B system. H atoms are omitted for the sake of clarity.

**Table 5:** DFT Gibbs Energies (Δ*G*) of the MEPs for (*S*)- and (*R*)-MeG Propagations Depending on the Assembly Mode

assembly mode	path	Δ*G*_RLS_^⧧^ (kcal mol^–1^)
**(*S*)-MeG Monomer**		
site B + B-chain	**M2-A** (*si*)	16.3 (TS1)
site B + A-chain	**M1-C** (*re*)	16.5 (TS1)
site A + A-chain	**M1-C** (*re*)	16.8 (TS1)
site A + B-chain	**M1-A** (*si*)	18.8 (TS2)
**(*R*)-MeG Monomer**		
site A + A-chain	**M2-A** (*re*)	16.0 (TS2)
site B + B-chain	**M2-A** (*re*)	16.7 (TS2)
site B + A-chain	**M1-C** (*si*)	16.7 (TS2)
site A + B-chain	**M2-A** (*re*)	17.9 (TS2)

The energetic profiles for the preferred mechanisms of the sequence-controlled synthesis of PLGA via ROP of 3-methyl glycolide at enantiopure Al-complex are reported in Figure 4. Note that the two MeG enantiomers prefer two opposite monomer enantiofaces (*si* for the (*S*)- and *re* for (*R*)-MeG), both characterized by the methyl substituents far from the ligand (see Figure 4).

**Figure 4:**
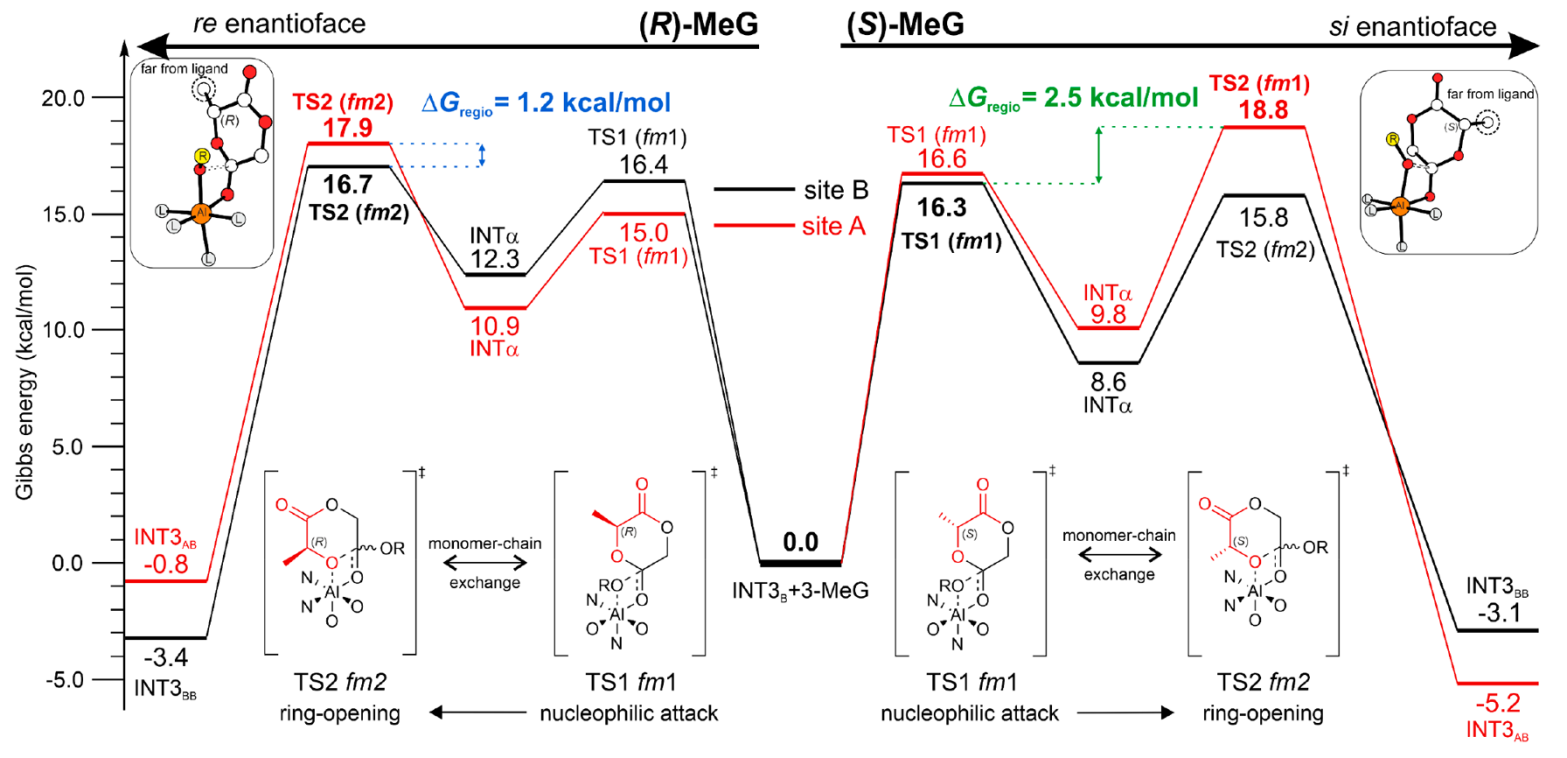
Minimum energy path for (*S*)-MeG (left) and (*R*)-MeG (right) insertions at (*R*)-**3B**. The paths deriving from site A and site B attacks for each monomer are reported in red and black, respectively, with the calculated regioselectivity.

In conclusion, despite the complexity of the mechanism paths and therefore the computational efforts reported here, interesting insights can be pointed out:

(1) The regioselectivity of alternate aliphatic polyesters obtained with (*R*) and (*S*) enantiomers of MeG is rationalized by computing all possible reaction paths displayed by the Al-chiral center. The (high) regioselectivity of (*S*)-MeG is attributed to preferential attack at site B with a **M2-A** mechanism characterized by TSs with *fac*-*mer* wrapping modes (*fm*1 for TS1 and *fm*2 for TS2). Occasional regiodefects (site A) are higher in energy due to an unfavorable ring-opening step (TS2). Comparison of the RLS for ring-opening at site A (TS2, *fm*1) and site B (TS1, *fm*1) reveal an energetic preference of B versus A-site attack of 2.5 kcal mol^–1^, in agreement with experimental results^14^ (Figure 4). Interestingly, a similar mechanism has already been suggested to explain the isotactic *rac*-LA^20^ and syndiotactic *meso*-LA polymerizations.^21^ Lower regioselectivity (1.2 kcal mol^–1^) is predicted for (*R*)-MeG, still with a **M2-A** mechanism but with a close similarity in the RLS (TS2, *fm*2) of sites A and B (Figure 4).
(2) The preferred reaction paths for the two MeG enantiomers display opposite monomer enantiofaces (*si* for (*S*) and *re* for (*R*); see Figure 4). Both enantiofaces are characterized by a longer distance of the methyl group bonded to the chiral C atom from the ligand framework (Figure S10 in the Supporting Information), revealing the role of the monomer enantioface, often neglected in stereoselective ROP.^24^
(3) The identification of the preferred reaction paths for (*S*)- and (*R*)-MeG allows a suitable catalyst ligand modification for enhancing the regioselectivity of both monomers.^15^ Future works on the ROP mechanisms of these interesting materials^25,26^ will be reported in due course.

## Supporting Information Available

Computational details (Figures S1–S10, Tables S1–S20) (PDF)
Cartesian coordinates of the MEPs structures mentioned in the text (XYZ)

## Supplementary Material

cs3c04955_si_001.pdf

cs3c04955_si_002.xyz

## References

[ref1] Samantaray P. K.; Little A.; Haddleton D. M.; McNally T.; Tan B.; Sun Z.; Huang W.; Ji Y.; Wan C. Poly(glycolic acid) (PGA): a versatile building block expanding high performance and sustainable Bioplastic applications. Green Chem. 2020, 22, 4055–4081. 10.1039/D0GC01394C.

[ref2] Garlotta D. A Literature Review of Poly(Lactic Acid). J. Polym. Environ. 2001, 9, 63–84. 10.1023/A:1020200822435.

[ref3] Rapier C. E.; Shea K. J.; Lee A. P. Investigating PLGA microparticle swelling behavior reveals an interplay of expansive intermolecular forces. Sci. Rep. 2021, 11, 14512 10.1038/s41598-021-93785-6. 34267274 PMC8282844

[ref4] Danhier F.; Ansorena E.; Silva J. M.; Coco R.; Le Breton A.; Préat V. PLGA-based nanoparticles: An overview of biomedical applications. J. Controlled Release 2012, 161, 505–522. 10.1016/j.jconrel.2012.01.043. 22353619

[ref5] Pan Z.; Ding J. Poly(lactide-*co*-glycolide) porous scaffolds for tissue engineering and regenerative medicine. Interface Focus 2012, 2, 366–377. 10.1098/rsfs.2011.0123. 23741612 PMC3363019

[ref6] Makadia H. K.; Siegel S. J. Poly Lactic-co-Glycolic Acid (PLGA) as Biodegradable Controlled Drug Delivery Carrier. Polymers 2011, 3, 1377–1397. 10.3390/polym3031377. 22577513 PMC3347861

[ref7] Wang Y.; Qin B.; Xia G.; Choi S. H. FDA’s Poly (Lactic-Co-Glycolic Acid) Research Program and Regulatory Outcomes. AAPS J. 2021, 23, 92 10.1208/s12248-021-00611-y. 34189655

[ref8] Dechy-Cabaret O.; Martin-Vaca B.; Bourissou D. Controlled Ring-Opening Polymerization of Lactide and Glycolide. Chem. Rev. 2004, 104, 6147–6176. 10.1021/cr040002s. 15584698

[ref9] Nifant’ev I. E.; Shlyakhtin A. V.; Bagrov V. V.; Komarov P. D.; Tavtorkin A. N.; Minyaev M. E.; Ivchenko P. V. Efficient synthetic approach to copolymers of glycolic and lactic acids for biomedical applications. Mendeleev Commun. 2018, 28, 412–414. 10.1016/j.mencom.2018.07.024.

[ref10] Brulé E.; Guo J.; Coates G. W.; Thomas C. M. Metal-Catalyzed Synthesis of Alternating Copolymers. Macromol. Rapid Commun. 2011, 32, 169–185. 10.1002/marc.201000524. 21433137

[ref11] Lutz J.-F.; Ouchi M.; Liu D. R.; Sawamoto M. Sequence-Controlled Polymers. Science 2013, 341, 1238149 10.1126/science.1238149. 23929982

[ref12] Dong C.-M.; Qiu K.-Y.; Gu Z.-W.; Feng X.-D. Synthesis of poly(D,L-lactic acid-*alt*-glycolic acid) from D,L-3-methylglycolide. J. Polym. Sci. A Polym. Chem. 2000, 38, 4179–4184. 10.1002/1099-0518(20001201)38:23<4179::AID-POLA20>3.0.CO;2-5.

[ref13] Takojima K.; Makino H.; Saito T.; Yamamoto T.; Tajima K.; Isono T.; Satoh T. An organocatalytic ring-opening polymerization approach to highly alternating copolymers of lactic acid and glycolic acid. Polym. Chem. 2020, 11, 6365–6373. 10.1039/D0PY01082K.

[ref14] Lu Y.; Swisher J. H.; Meyer T. Y.; Coates G. W. Chirality-Directed Regioselectivity: An Approach for the Synthesis of Alternating Poly(Lactic-*co*-Glycolic Acid). J. Am. Chem. Soc. 2021, 143, 4119–4124. 10.1021/jacs.1c00248. 33687202

[ref15] Lu Y.; Coates G. W. Pairing-Enhanced Regioselectivity: Synthesis of Alternating Poly(lactic-*co*-glycolic acid) from Racemic Methyl-Glycolide. J. Am. Chem. Soc. 2023, 145, 22425–22432. 10.1021/jacs.3c05941. 37793193

[ref16] Spassky N.; Wisniewski M.; Pluta C.; Le Borgne A. Highly stereoelective polymerization of *rac*-(d,l)-lactide with a chiral Schiff’s base/aluminium alkoxide initiator. Macromol. Chem. Phys. 1996, 197, 2627–2637. 10.1002/macp.1996.021970902.

[ref17] Wang X.; Huang Y.; Xie X.; Liu Y.; Huo Z.; Lin M.; Xin H.; Tong R. Bayesian-optimization-assisted discovery of stereoselective aluminum complexes for ring-opening polymerization of racemic lactide. Nat. Commun. 2023, 14, 3647 10.1038/s41467-023-39405-5. 37339991 PMC10282063

[ref18] Xie X.; Huo Z.; Jang E.; Tong R. Recent advances in enantioselective ring-opening polymerization and copolymerization. Commun. Chem. 2023, 6 (1), 202 10.1038/s42004-023-01007-z. 37775528 PMC10541874

[ref19] Ovitt T. M.; Coates G. W. Stereoselective Ring-Opening Polymerization of *meso*-Lactide: Synthesis of Syndiotactic Poly(lactic acid). J. Am. Chem. Soc. 1999, 121, 4072–4073. 10.1021/ja990088k.

[ref20] D’Alterio M. C.; De Rosa C.; Talarico G. Stereoselective Lactide Polymerization: The Challenge of Chiral Catalyst Recognition. ACS Catal. 2020, 10, 2221–2225. 10.1021/acscatal.9b05109.

[ref21] D’Alterio M. C.; De Rosa C.; Talarico G. Syndiotactic PLA from *meso*-LA polymerization at the Al-chiral complex: a probe of DFT mechanistic insights. Chem. Commun. 2021, 57, 1611–1614. 10.1039/D0CC07787A. 33447839

[ref22] Falivene L.; Barone V.; Talarico G. Unraveling the role of entropy in tuning unimolecular *vs*. bimolecular reaction rates: The case of olefin polymerization catalyzed by transition metals. Mol. Catal. 2018, 452, 138–144. 10.1016/j.mcat.2018.04.012.

[ref23] Falivene L.; Cavallo L.; Talarico G. Buried Volume Analysis for Propene Polymerization Catalysis Promoted by Group 4 Metals: A Tool for Molecular Mass Prediction. ACS Catal. 2015, 5, 6815–6822. 10.1021/acscatal.5b01363.

[ref24] Yuntawattana N.; McGuire T. M.; Durr C. B.; Buchard A.; Williams C. K. Indium phosphasalen catalysts showing high isoselectivity and activity in racemic lactide and lactone ring opening polymerizations. Catal. Sci. Technol. 2020, 10, 7226–7239. 10.1039/D0CY01484B.

[ref25] Han J. W.; Hollmann F.; Luque R.; Song I. K.; Talarico G.; Tatsumi T.; Yan N. Molecular Catalysis for the Chemistry of the future: a perspective. Mol. Catal. 2022, 522, 112233 10.1016/j.mcat.2022.112233.

[ref26] Coates G. W.; Getzler Y. D. Y. L. Chemical recycling to monomer for an ideal, circular polymer economy. Nat. Rev. Mater. 2020, 5, 501–516. 10.1038/s41578-020-0190-4.

