# Supplementary material for: Mechanism of Alternating Poly(lactic-co-glycolic acid) Formation by Polymerization of (S)- and (R)-3-Methyl Glycolide Using an Enantiopure Aluminum Complex
Source: ACS Catal. 2023 Dec 18;14(1):318–23. doi: 10.1021/acscatal.3c04955 (PMC10775139; doi:10.1021/acscatal.3c04955)
Supplement: Supplementary file 1 — cs3c04955_si_001.pdf [file cs3c04955_si_001.pdf]

SUPPORTING INFORMATION

Mechanism of Alternating Poly(lactic-*co*-glycolic acid) Formation by Polymerization of (*S*)- and (*R*)-3-Methyl Glycolide using an Enantiopure Aluminum Complex

Yolanda Rusconi,[1,2] Massimo Christian D'Alterio,[2] Claudio De Rosa,[2*] Yiye Lu,[3] Sarah M. Severson,[3] Geoffrey W. Coates,[3] and Giovanni Talarico[1,2*]

[1] Scuola Superiore Meridionale, Largo San Marcellino, 80138 Napoli, Italy

[2] Dipartimento di Scienze Chimiche, Università degli Studi di Napoli Federico II, 80126 Napoli, Italy

[3] Department of Chemistry and Chemical Biology, Baker Laboratory, Cornell University, Ithaca, NY 14853-1301, USA

Corresponding authors: claudio.derosa@unina.it; talarico@unina.it

Table of contents

Details of computational methods	S2
Figures S1-S2	S3
Figure S3	S4
Figure S4	S5
Figure S5	S6
Figures S6-S7	S7
Figures S8-S9	S8
Figure S10	S9
Tables S1-S2	S10
Tables S3-S4	S11
Tables S5-S7	S12
Tables S8-S9	S13
Table S10	S14
Tables S11-S12	S15
Table S13	S16
Table S14	S17
Table S15	S18
Tables S16-S17	S19
Table S18	S20
Table S19	S21
Table S20	S22
References	S23

1. Details of computational methods

All geometry optimizations were performed using the Gaussian09 set of programs[1], using the B3LYP functional[2,3] in conjunction with two different layers of basis set: 6-311G(d,p)[4,5] for the Al center to better represent the coordination geometry and the polarized split valence SVP[6] for H, C, N and O. Stationary points were characterized using vibrational analysis, and this analysis has been also used to calculate zero-point energies and thermal (enthalpy and entropy) corrections (298.15 K, 1 bar). An improvement of the electronic energies was obtained through single-point energy calculations using a 6-311G(d,p) basis set on all atoms. In this calculation, dispersion corrections were performed using EmpiricalDispersion=GD3BJ[7] of Grimme with Becke-Johnson damping in the G09.E01 package. These energies added to the thermal corrections calculated at the SVP-level and the solvation contribution (toluene or dichloromethane, DCM) calculated with PCM and CPCM models,[8,9] respectively, are named ΔG. This computational approach has been used after a benchmark with the experimental results achieved on stereoselective ROP polymerization by *rac*-lactide (LA)[10] and *meso*-LA[11] promoted by the same catalytic system used in this work and on stereoselective olefin polymerization transition metal catalyzed.[12,13] Finally, the calculated regioselectivities reported in the main text (2.5 and 1.2 kcal for (*S*) and (*R*)-MeG, respectively) are in good agreement with the experimental regioselectivities of 96% and 78% of the polymers synthesized in DCM as estimated by ^{1}H NMR spectra on methine (δ = 5.2–5.3 ppm) and methylene (δ = 4.6–4.9 ppm) regions reported in a recent paper.[14] A similar trend for the regioselectivities was also found by changing the functional (see e.g. M06[15] and ωB97XD[16] results reported in Tables S7 and S18). Overall, we found that the use of dispersion correction[7] is important for the energetic comparison with the experimental data although a minimal effect has been observed on the geometry optimizations.

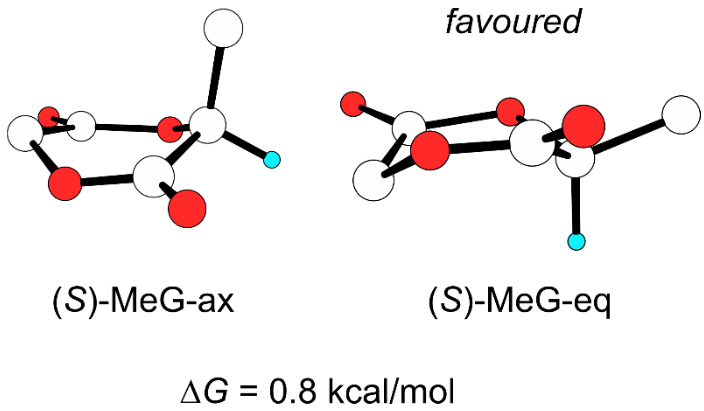

Figure S1. Conformations of (*S*)-methyl glycolide with the methyl substituent in axial (right) or equatorial (right) position.

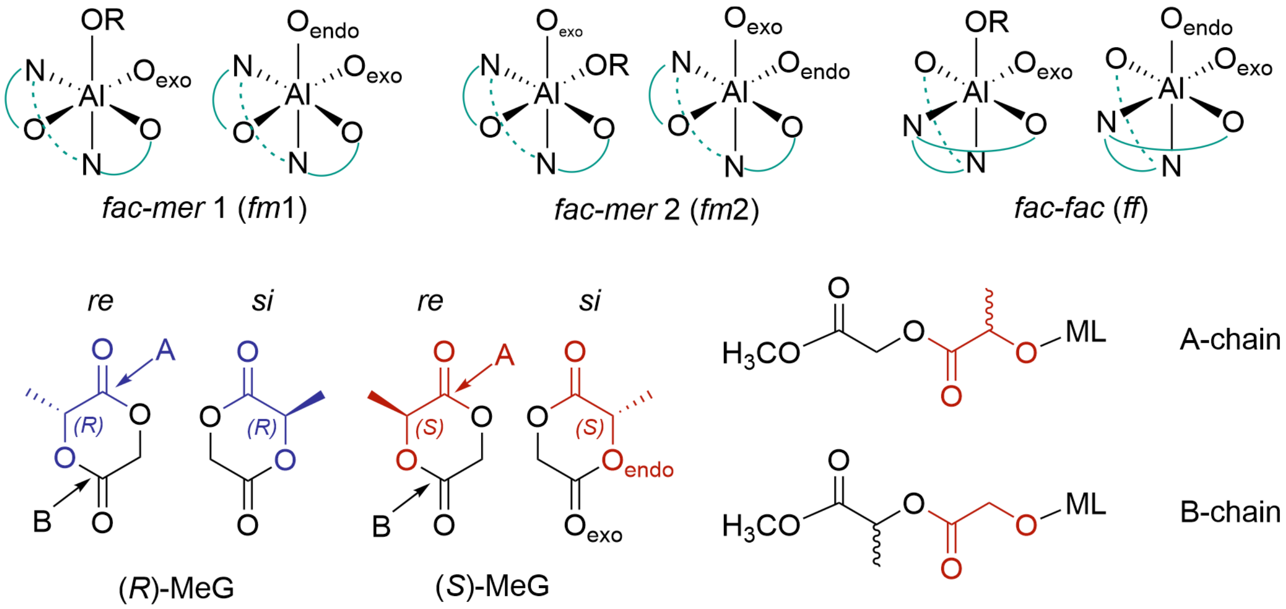

Figure S2. Elements of chirality: at the top, the possible conformations of TS1 and TS2, where OR = OMe, O^{i}Pr or A- or B-chain and O_{endo} and O_{exo} = endocyclic and exocyclic O of MeG. At the bottom left, the two enantiomers of MeG ((*R*)-MeG and (*S*)-MeG) with the two enantiofaces (*re* and *si*) and the two attack sites A and B; at the bottom-right, the chirality of the two growing-chains. The LA portion of MeG is represented in blue for (*R*)-MeG and in red for (*S*)-MeG, while the GA portion of MeG is represented in in black.

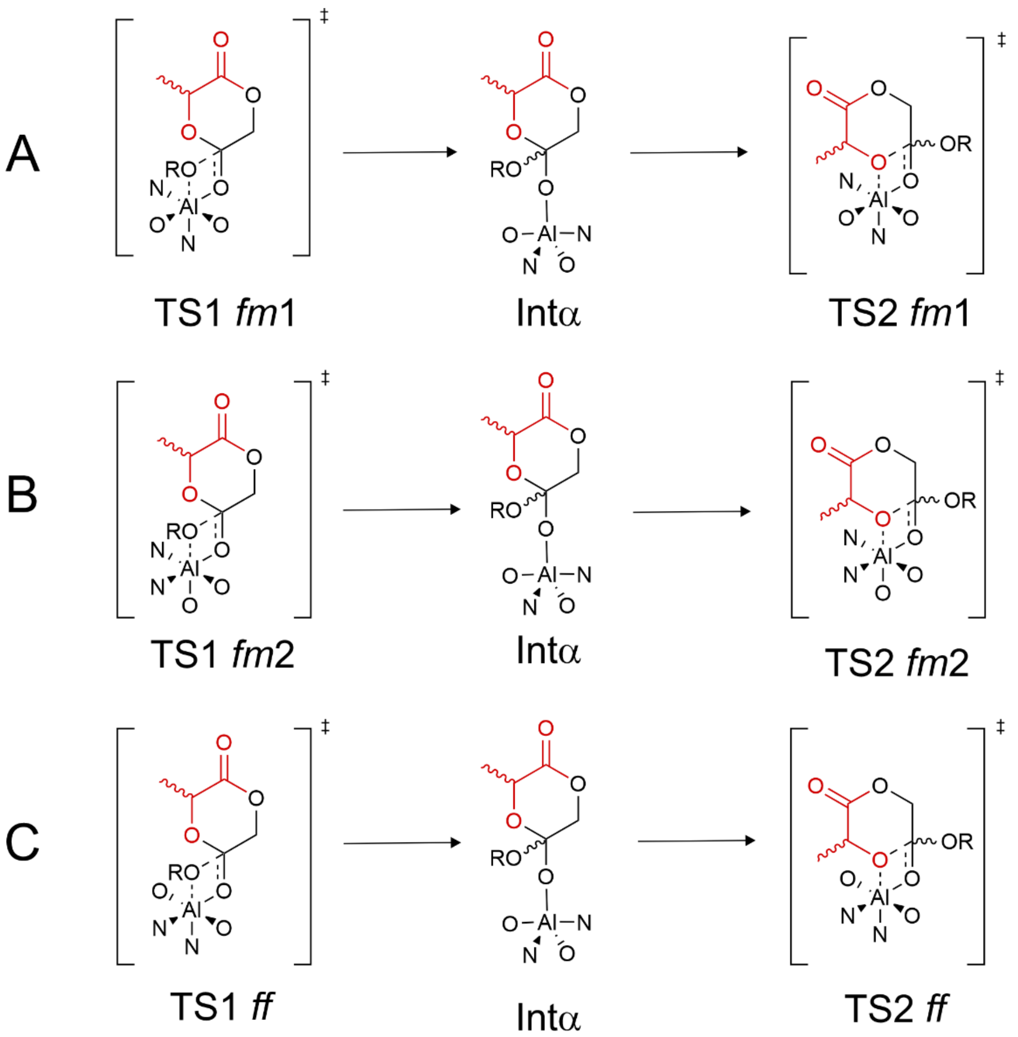

Figure S3. The three different paths computed for mechanism 1 (**M1**). The attack on site A follows the same mechanisms, but for simplicity only the attack at site B is reported.

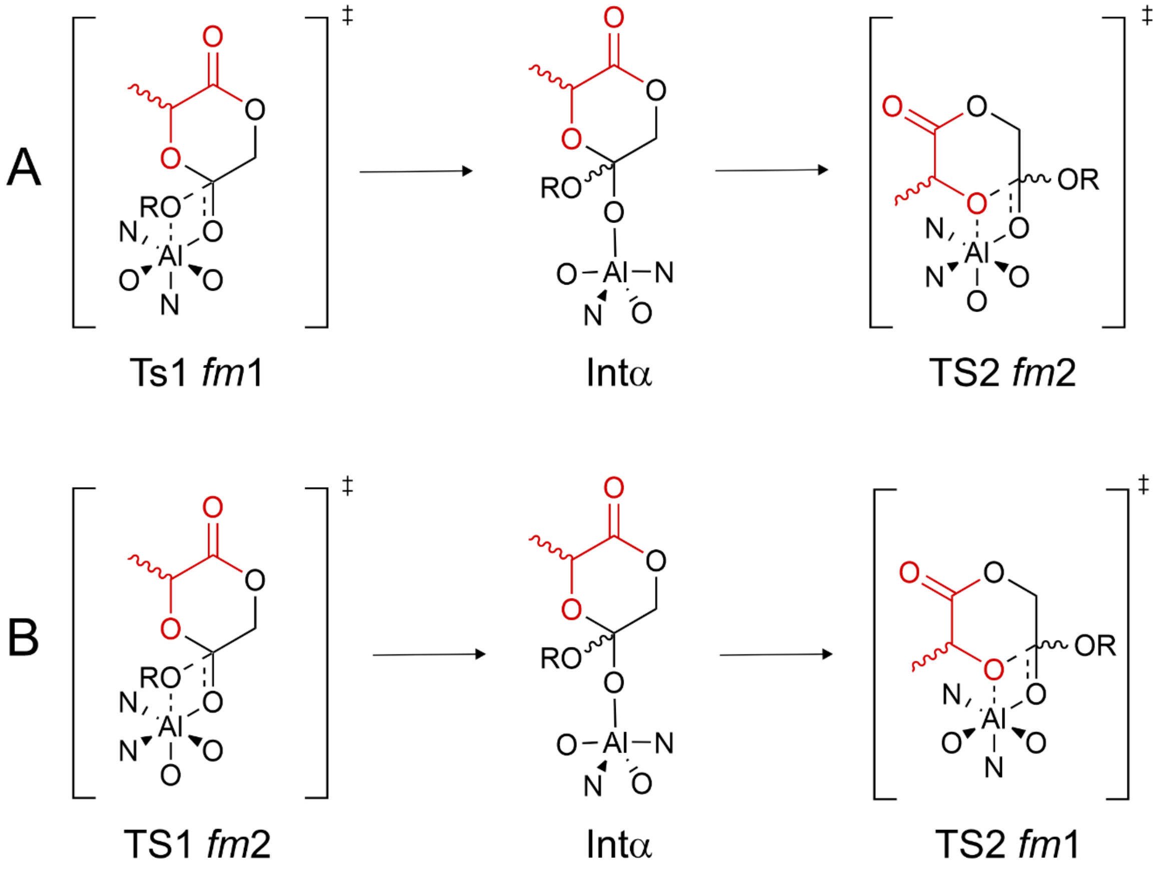

Figure S4. The two different paths computed for mechanism 2 (**M2**). The attack on site A follows the same mechanisms, but for simplicity only the attack at site B is reported.

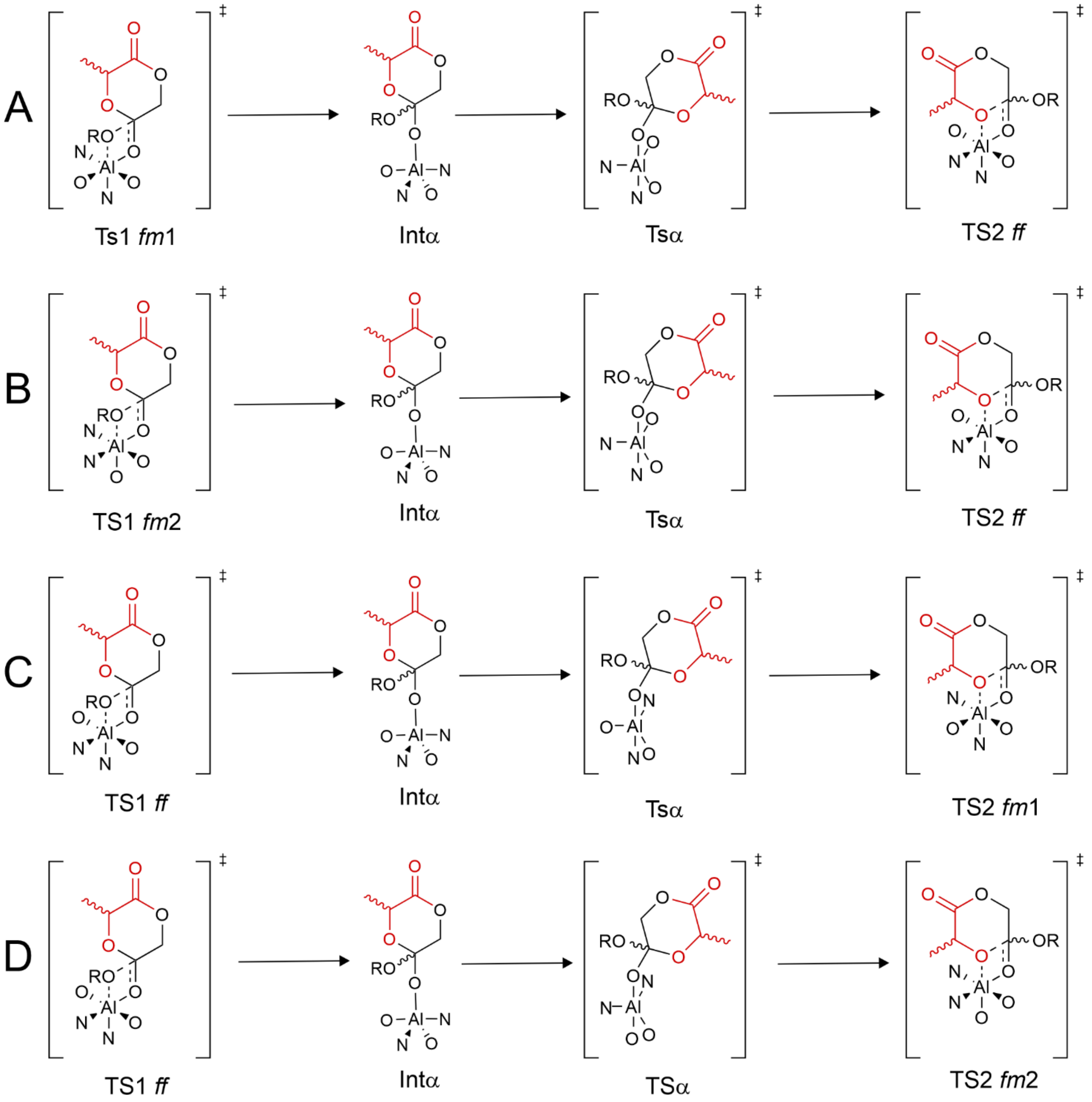

Figure S5. The four different paths computed for mechanism 3 (**M3**). The attack on site A follows the same mechanisms, but for simplicity only the attack at site B is reported.

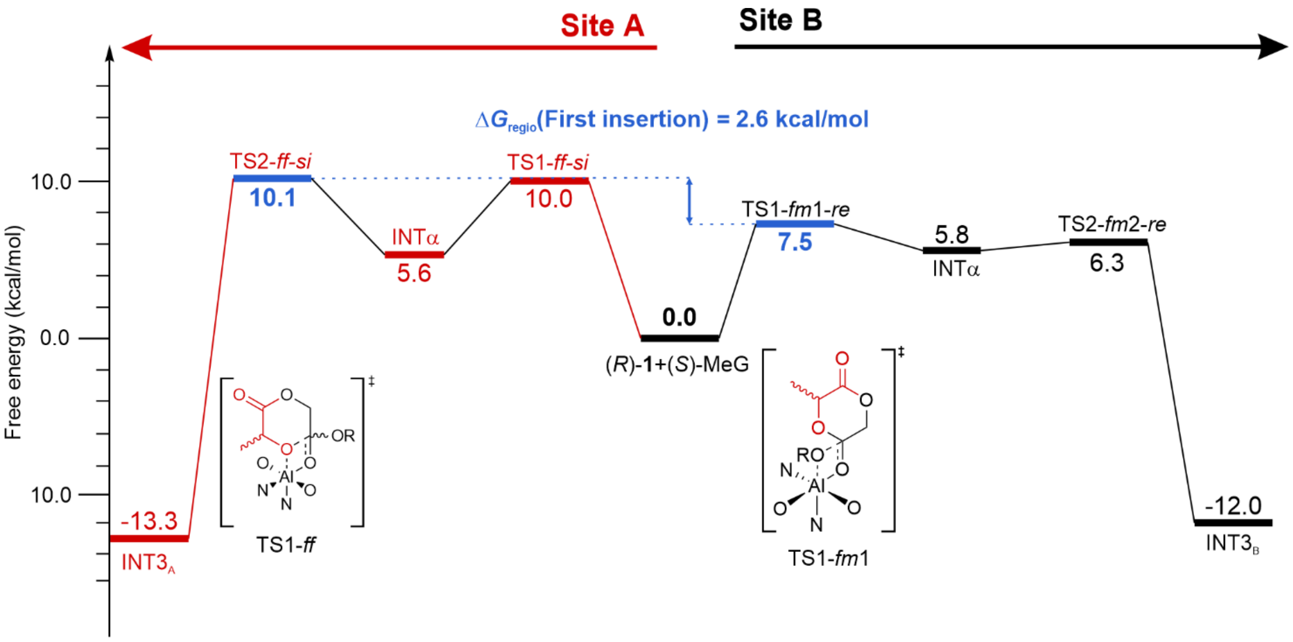

Figure S6. Gibbs energies for the minimum energy path for the (*S*)-MeG insertion into the Al-OCH_3 bond at site A (left) and B (right). The same value has been obtained by computing the electronic energies (ΔE_{regio} = 2.6 kcal/mol).

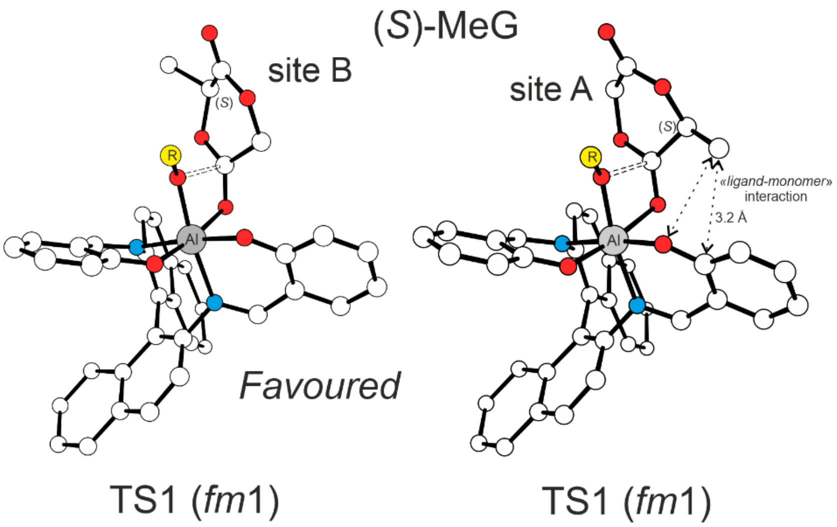

Figure S7. DFT geometries of the TS1 arising from the insertion of (*S*)-MeG in the Al-OCH_3 bond at site B (left) and A (right). Hydrogen atoms are omitted for clarity and distances in Å.

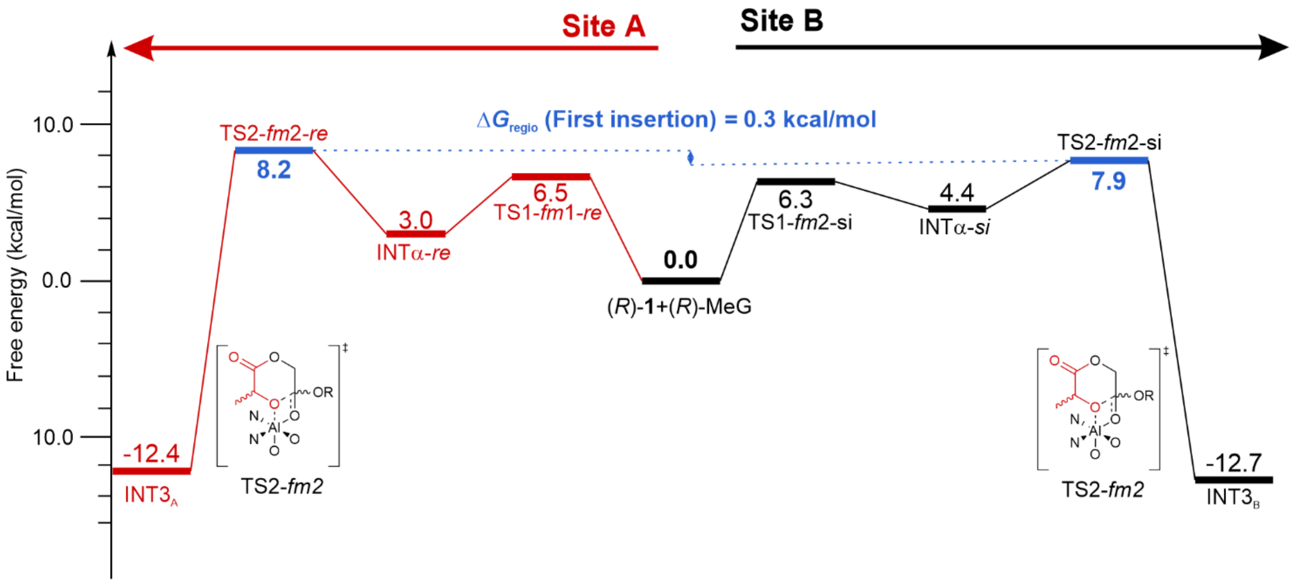

Figure S8. Minimum energy path for the (*R*)-MeG insertion into the Al-OCH3 bond at site A (left) and B (right).

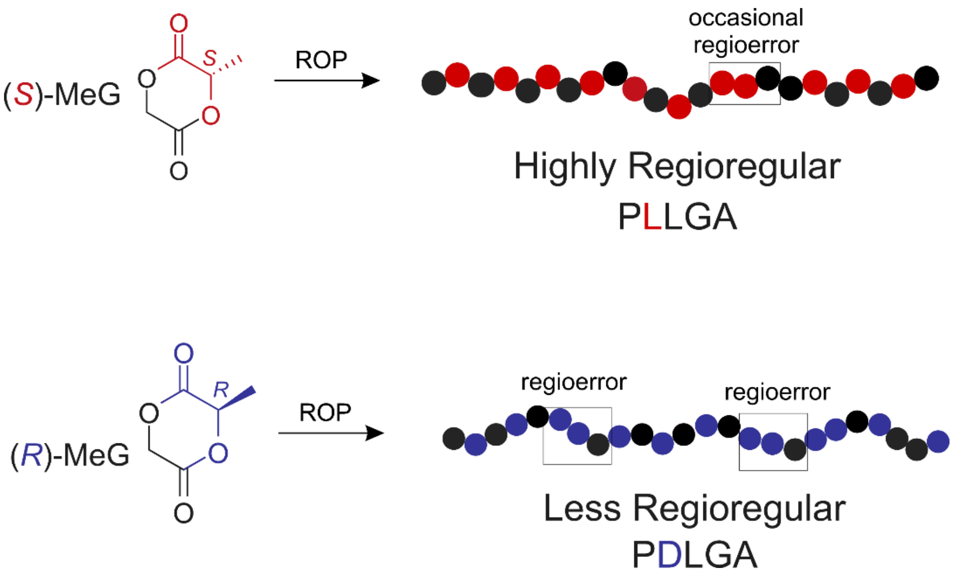

Figure S9. Schematic representation of the polymer microstructure.

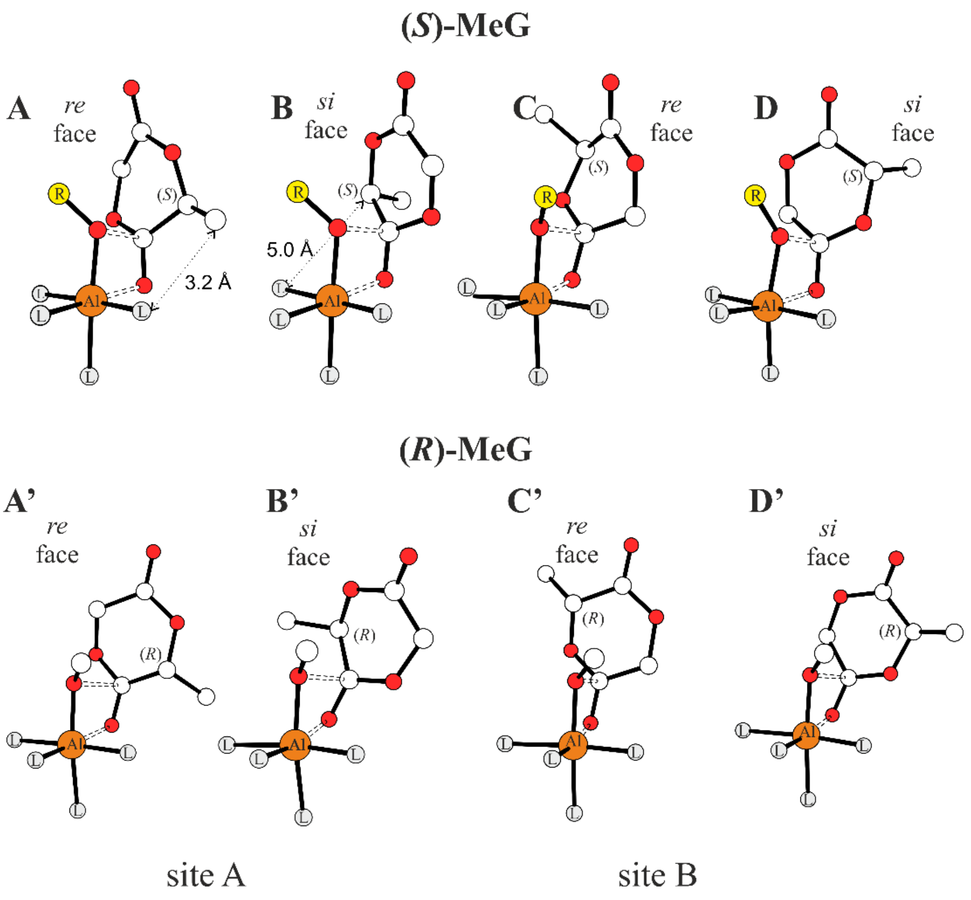

Figure S10. Schematic 3D representation of the possible attacks on *re* and *si* enantiofaces.

Table S1. TSs Gibbs energies (Δ*G*, with respect to (*R*)-1 + monomer in kcal/mol) for all reaction paths computed for (*S*)-MeG insertion into the Al-OCH_3 bond at site A. Values calculated in toluene (dichloromethane, DCM, reported in brackets). In bold red and green the low-lying paths in toluene and DCM, respectively.

Monomer	*S*				Monomer	*S*			
Site	A				Site	A			
Enantioface	*re*				Enantioface	*si*			
		**TS1**		**TS2**			**TS1**		**TS2**
Mechanism 1 (**M1**)	A	9.6 (10.7)		7.9 (9.2)	Mechanism 1 (**M1**)	A	6.5 (7.4)		11.9 (13.2)
	B	12.1 (12.9)		3.4 (5.0)		B	5.1 (6.6)		11.4 (13.6)
	C	9.0 (10.5)		3.7 (5.9)		C	**8.2** **(10.0)**		**8.4** **(10.1)**
		**TS1**		**TS2**			**TS1**		**TS2**
Mechanism 2 (**M2**)	A	9.6 (10.7)		3.4 (5.0)	Mechanism 2 (**M2**)	A	6.5 (7.4)		11.4 (13.6)
	B	12.1 (12.9)		7.9 (9.2)		B	5.1 (6.6)		11.9 (13.2)
		**TS1**	**TSα**	**TS2**			**TS1**	**TSα**	**TS2**
Mechanism 3 (**M3**)	A	9.6 (10.7)	12.4 (14.7)	3.7 (5.9)	Mechanism 3 (**M3**)	A	6.5 (7.4)	11.0 (13.0)	8.4 (10.1)
	B	12.1 (12.9)	12.4 (14.7)	3.7 (5.9)		B	5.1 (6.6)	11.0 (13.0)	8.4 (10.1)
	C	9.0 (10.5)	12.4 (14.7)	7.9 (9.2)		C	8.2 (10.0)	11.0 (13.0)	11.9 (13.2)
	D	9.0 (10.5)	12.4 (14.7)	3.4 (5.0)		D	8.2 (10.0)	11.0 (13.0)	11.4 (13.6)

Table S2. TSs Gibbs energies (Δ*G*, with respect to (*R*)-1 + monomer in kcal/mol) for all reaction paths computed for (*S*)-MeG insertion into the Al-OCH_3 bond at site B. Values calculated in toluene (dichloromethane, DCM, reported in brackets). In bold red and green the low-lying paths in toluene and DCM, respectively.

Monomer	*S*				Monomer	*S*			
Site	B				Site	B			
Enantioface	*re*				Enantioface	*si*			
		**TS1**		**TS2**			**TS1**		**TS2**
Mechanism 1 (**M1**)	A	6.2 (7.5)		8.1	Mechanism 1 (**M1**)	A	7.7		9.2
	B	8.1		4.8 (6.3)		B	5.7 (7.1)		7.0 (8.9)
	C	8.0		4.7		C	8.9		7.7 (10.0)
		**TS1**		**TS2**			**TS1**		**TS2**
Mechanism 2 (**M2**)	A	**6.2** **(7.5)**		**4.8** **(6.3)**	Mechanism 2 (**M2**)	A	7.7		7.0 (8.9)
	B	8.1		8.1		B	5.7 (7.1)		9.2
		**TS1**	**TSα**	**TS2**			**TS1**	**TSα**	**TS2**
Mechanism 3 (**M3**)	A	6.2 (7.5)	11.8 (14.2)	4.7	Mechanism 3 (**M3**)	A	7.7	10.1 (11.9)	7.7 (10.0)
	B	8.1	11.8 (14.2)	4.7		B	5.7 (7.1)	10.1 (11.9)	7.7 (10.0)
	C	8.0	11.8 (14.2)	8.1		C	8.9	10.1 (11.9)	9.2
	D	8.0	11.8 (14.2)	4.8 (6.3)		D	8.9	10.1 (11.9)	7.0 (8.9)

Table S3. DFT Electronic Energies (Δ*E*, in kcal·mol^{-1}) and Gibbs Energies (in brackets) ((Δ*G*), in kcal·mol^{-1}) of the MEPs for (*S*)-MeG Insertion promoted by (*R*)-1. Preferred Paths are Reported in Bold.

Site	Path	Wrapping mode **TS1-TS2**	**TS1** Δ*E* (Δ*G*)	**TSα** Δ*E* (Δ*G*)	**TS2** Δ*E* (Δ*G*)
**A**	**M1-C**	***ff-ff* (*si*)**	**-5.9 (10.0)**		**-6.6 (10.1)**
	M2-A	*fm*1-*fm*2 (*re*)	-4.9 (10.7)		-11.7 (5.0)
	M3-B	*fm*2-*ff* (*si*)	-9.5 (6.6)	-5.1 (13.0)	-6.6 (10.1)
**B**	M1-B	*fm*2-*fm*2 (*si*)	-8.9 (7.1)		-8.4 (8.9)
	**M2-A**	***fm*1-*fm*2 (*re*)**	**-8.5 (7.5)**		**-11.3 (6.3)**
	M3-B	*fm*2-*ff* (*si*)	-8.9 (7.1)	-5.4 (11.9)	-6.7 (10.0)

Table S4. TSs Gibbs energies (Δ*G*, with respect to (*R*)-1 + monomer in kcal/mol) for all reaction paths computed for (*R*)-MeG insertion into the Al-OCH_3 bond at site A. Values calculated in toluene (dichloromethane, DCM, reported in brackets). In bold red and green the low-lying paths in toluene and DCM, respectively.

Monomer<br>Site<br>Enantioface	*R*<br>A<br>*re*				Monomer<br>Site<br>Enantioface	*R*<br>A<br>*si*			
		**TS1**		**TS2**			**TS1**		**TS2**
Mechanism 1 (**M1**)	A	4.5 (6.5)		7.0 (9.0)	Mechanism 1 (**M1**)	A	11.9		8.0
	B	8.5		7.2 (8.2)		B	7.6 (8.8)		5.0
	C	8.4		4.5		C	10.7		5.1 (7.4)
		**TS1**		**TS2**			**TS1**		**TS2**
Mechanism 2 (**M2**)	A	**4.5** **(6.5)**		**7.2** **(8.2)**	Mechanism 2 (**M2**)	A	11.9		5.0
	B	8.5		7.0 (9.0)		B	7.6 (8.8)		8.0
		**TS1**	**TSα**	**TS2**			**TS1**	**TSα**	**TS2**
Mechanism 3 (**M3**)	A	4.5 (6.5)	9.3 (11.7)	4.5	Mechanism 3 (**M3**)	A	11.9	8.4 (10.3)	5.1 (7.4)
	B	8.5	9.3 (11.7)	4.5		B	7.6 (8.8)	8.4 (10.3)	5.1 (7.4)
	C	8.4	9.3 (11.7)	7.0 (9.0)		C	10.7	8.4 (10.3)	8.0
	D	8.4	9.3 (11.7)	7.2 (8.2)		D	10.7	8.4 (10.3)	5.0

Table S5. TSs Gibbs energies (Δ*G*, with respect to (*R*)-1 + monomer in kcal/mol) for all reaction paths computed for (*R*)-MeG insertion into the Al-OCH_3 bond at site B. Values calculated in toluene (dichloromethane, DCM, reported in brackets). In bold red and green the low-lying paths in toluene and DCM, respectively.

Monomer<br>Site<br>Enantioface	*R*<br>B<br>*re*				Monomer<br>Site<br>Enantioface	*R*<br>B<br>*si*			
		**TS1**		**TS2**			**TS1**		**TS2**
Mechanism 1 (**M1**)	A	6.0 (7.9)		7.8 (9.5)	Mechanism 1 (**M1**)	A	6.9 (7.5)		9.4
	B	9.0		8.0		B	**5.0 (6.3)**		**6.2 (7.9)**
	C	8.5		5.5 (7.6)		C	9.5		5.2
		**TS1**		**TS2**			**TS1**		**TS2**
Mechanism 2 (**M2**)	A	6.0 (7.9)		8.0	Mechanism 2 (**M2**)	A	6.9 (7.5)		6.2 (7.9)
	B	9.0		7.8 (9.5)		B	5.0 (6.3)		9.4
		**TS1**	**TSα**	**TS2**			**TS1**	**TSα**	**TS2**
Mechanism 3 (**M3**)	A	6.0 (7.9)	9.5 (11.4)	5.5 (7.6)	Mechanism 3 (**M3**)	A	6.9 (7.5)	11.2 (12.9)	5.2
	B	9.0	9.5 (11.4)	5.5 (7.6)		B	5.0 (6.3)	11.2 (12.9)	5.2
	C	8.5	9.5 (11.4)	7.8 (9.5)		C	9.5	11.2 (12.9)	9.4
	D	8.5	9.5 (11.4)	8.0		D	9.5	11.2 (12.9)	6.2 (7.9)

Table S6. DFT Electronic Energies (Δ*E*, in kcal·mol^{-1}) and Gibbs Energies (in brackets) ((Δ*G*), in kcal·mol^{-1}) of the MEPs for (*R*)-MeG Insertion promoted by (*R*)-1. Preferred Paths are Reported in Bold.

Site	Path	Wrapping mode of **TS1-TS2**	**TS1**<br>Δ*E* (Δ*G*)	**TSα**<br>Δ*E* (Δ*G*)	**TS2**<br>Δ*E* (Δ*G*)
**A**	M1-A	*fm*1-*fm*1 (*re*)	-8.5 (6.5)		-9.1 (9.0)
	**M2-B**	***fm*1-*fm*2 (*re*)**	**-8.5 (6.5)**		**-8.6 (8.2)**
	M3-B	*fm*2-*ff* (*si*)	-7.7 (8.8)	-7.4 (10.3)	-9.6 (7.4)
**B**	**M1-B**	***fm*2-*fm*2 (*si*)**	**-9.9 (6.3)**		**-9.2 (7.9)**
	M2-A	*fm*1-*fm*2 (*si*)	-7.5 (7.5)		-9.2 (7.9)
	M3-B	*fm*2-*ff* (*re*)	-7.8 (7.9)	-6.0 (11.4)	-10.5 (7.6)

Table S7. Electronic Energies (ΔΔ*E*) and Gibbs Energies (in brackets) ((ΔΔ*G*) in kcal·mol^{-1} calculated for the regioselectivity of the (*S*)-MeG and (*R*)-MeG insertions promoted by (*R*)-1 with different functionals.

Monomer	Preferred site	SP methodology		
		B3LYP-D3(BJ)<br>ΔΔ*E* (ΔΔ*G*)	M06<br>ΔΔ*E* (ΔΔ*G*)	*ω*B97XD<br>ΔΔ*E* (ΔΔ*G*)
**(*S*)-MeG**	B	1.9 (2.6)	3.0 (3.6)	1.7 (2.2)
**(*R*)-MeG**	B	0.6 (0.3)	0.7 (0.4)	1.3 (1.1)

Table S8. TSs Gibbs energies (Δ*G*, with respect to (*R*)-2 + monomer in kcal/mol) for all reaction paths computed for (*S*)-MeG insertion into the Al-O^{i}Pr bond at site A. Values calculated in toluene (dichloromethane, DCM, reported in brackets). In bold red and green the low-lying paths in toluene and DCM, respectively.

Monomer<br>Site<br>Enantioface	*S*<br>A<br>*re*				Monomer<br>Site<br>Enantioface	*S*<br>A<br>*si*			
		**TS1**		**TS2**			**TS1**		**TS2**
Mechanism 1 (**M1**)	A	13.0 (14.2)		8.6 (9.9)	Mechanism 1 (**M1**)	A	10.3 **(11.6)**		12.6 **(13.8)**
	B	19.4 (20.5)		4.6 (6.1)		B	13.9 (15.7)		12.5 (14.6)
	C	**12.4** (14.3)		**8.5** (10.7)		C	12.5 (14.3)		9.3 (11.1)
		**TS1**		**TS2**			**TS1**		**TS2**
Mechanism 2 (**M2**)	A	13.0 (14.2)		4.6 (6.1)	Mechanism 2 (**M2**)	A	**10.3** (11.6)		**12.5** (14.6)
	B	19.4 (20.5)		8.6 (9.9)		B	13.9 (15.7)		12.6 (13.8)
		**TS1**	**TSα**	**TS2**			**TS1**	**TSα**	**TS2**
Mechanism 3 (**M3**)	A	13.0 (14.2)	13.4 (15.7)	8.5 (10.7)	Mechanism 3 (**M3**)	A	10.3 (11.6)	14.0 (16.0)	9.3 (11.1)
	B	19.4 (20.5)	13.4 (15.7)	8.5 (10.7)		B	13.9 (15.7)	14.0 (16.0)	9.3 (11.1)
	C	12.4 (14.3)	13.4 (15.7)	8.6 (9.9)		C	12.5 (14.3)	14.0 (16.0)	12.6 (13.8)
	D	12.4 (14.3)	13.4 (15.7)	4.6 (10.7)		D	12.5 (14.3)	14.0 (16.0)	12.5 (14.6)

Table S9. TSs Gibbs energies (Δ*G*, with respect to (*R*)-2 + monomer in kcal/mol) for all reaction paths computed for the (*S*)-MeG insertion into the Al-O^{i}Pr bond at site B. Values calculated in toluene (dichloromethane, DCM, reported in brackets). In bold red and green the low-lying paths in toluene and DCM, respectively.

Monomer<br>Site<br>Enantioface	*S*<br>B<br>*re*				Monomer<br>Site<br>Enantioface	*S*<br>B<br>*si*			
		**TS1**		**TS2**			**TS1**		**TS2**
Mechanism 1 (**M1**)	A	8.5 (10.0)		8.8 (10.2)	Mechanism 1 (**M1**)	A	11.3 (12.6)		10.2
	B	16.1		6.1 (7.6)		B	14.0		7.8
	C	10.7 (12.2)		5.4 (7.4)		C	13.0		8.5 (10.7)
		**TS1**		**TS2**			**TS1**		**TS2**
Mechanism 2 (**M2**)	A	**8.5** **(10.0)**		**6.1** **(7.6)**	Mechanism 2 (**M2**)	A	11.3 (12.6)		7.8
	B	16.1		8.8 (10.2)		B	14.0		10.2
		**TS1**	**TSα**	**TS2**			**TS1**	**TSα**	**TS2**
Mechanism 3 (**M3**)	A	8.5 (10.0)	12.6 (14.9)	5.4 (7.4)	Mechanism 3 (**M3**)	A	11.3 (12.6)	12.5 (14.1)	8.5 (10.7)
	B	16.1	12.6 (14.9)	5.4 (7.4)		B	14.0	12.5 (14.1)	8.5 (10.7)
	C	10.7 (12.2)	12.6 (14.9)	8.8 (10.2)		C	13.0	12.5 (14.1)	10.2
	D	10.7 (12.2)	12.6 (14.9)	6.1 (7.6)		D	13.0	12.5 (14.1)	7.8

Table S10. TSs Gibbs energies (Δ*G*, with respect to (*R*)-3B + monomer in kcal/mol) for all reaction paths computed for the (*S*)-MeG insertion into the Al-B-chain bond at site B. Values calculated in toluene (dichloromethane, DCM, reported in brackets). In bold red and green the low-lying paths in toluene and DCM, respectively.

Monomer	*S*				Monomer	*S*			
Site	B				Site	B			
Chain	B				Chain	B			
Enantioface	*re*				Enantioface	*si*			
		**TS1**		**TS2**			**TS1**		**TS2**
Mechanism 1 (**M1**)	A	12.0 (14.5)		15.5 (17.8)	Mechanism 1 (**M1**)	A	14.0 (16.3)		17.3 (19.2)
	B	19.6 (22.3)		14.8 (17.3)		B	17.2 (20.1)		14.3 (15.8)
	C	18.1 (20.5)		13.0 (15.7)		C	20.2 (22.0)		16.0 (18.2)
		**TS1**		**TS2**			**TS1**		**TS2**
Mechanism 2 (**M2**)	A	12.0 (14.5)		14.8 (17.3)	Mechanism 2 (**M2**)	A	**14.0** **(16.3)**		**14.3** **(15.8)**
	B	19.6 (22.3)		15.5 (17.8)		B	17.2 (20.1)		17.3 (19.2)
		**TS1**	**TSα**	**TS2**			**TS1**	**TSα**	**TS2**
Mechanism 3 (**M3**)	A	12.0 (14.5)	16.8 (20.1)	13.0 (15.7)	Mechanism 3 (**M3**)	A	14.0 (16.3)	15.1 (17.5)	16.0 (18.2)
	B	19.6 (22.3)	16.8 (20.1)	13.0 (15.7)		B	17.2 (20.1)	15.1 (17.5)	16.0 (18.2)
	C	18.1 (20.5)	16.8 (20.1)	15.5 (17.8)		C	20.2 (22.0)	15.1 (17.5)	17.3 (19.2)
	D	18.1 (20.5)	16.8 (20.1)	14.8 (17.3)		D	20.2 (22.0)	15.1 (17.5)	14.3 (15.8)

Table S11. TSs Gibbs energies (Δ*G*, with respect to (*R*)-3B + monomer in kcal/mol) for all reaction paths computed for the (*S*)-MeG insertion into the Al-B-chain bond at site A. Values calculated in toluene (dichloromethane, DCM, reported in brackets). In bold red and green the low-lying paths in toluene and DCM, respectively.

Monomer	*S*				Monomer	*S*			
Site	A				Site	A			
Chain	B				Chain	B			
Enantioface	*re*				Enantioface	*si*			
		**TS1**		**TS2**			**TS1**		**TS2**
Mechanism 1 (**M1**)	A	18.7 (21.6)		16.8 (18.9)	Mechanism 1 (**M1**)	A	**14.2** **(16.6)**		**17.8** **(18.8)**
	B	24.0 (26.8)		12.0 (14.2)		B	17.1 (20.1)		18.7 (20.9)
	C	20.1 (23.3)		12.0 (14.3)		C	17.1 (20.1)		18.7 (20.7)
		**TS1**		**TS2**			**TS1**		**TS2**
Mechanism 2 (**M2**)	A	18.7 (21.6)		12.0 (14.2)	Mechanism 2 (**M2**)	A	14.2 (16.6)		18.7 (20.9)
	B	24.0 (26.8)		16.8 (18.9)		B	17.1 (20.1)		17.8 (18.8)
		**TS1**	**TSα**	**TS2**			**TS1**	**TSα**	**TS2**
Mechanism 3 (**M3**)	A	18.7 (21.6)	14.9 (17.7)	12.0 (14.3)	Mechanism 3 (**M3**)	A	14.2 (16.6)	22.0 (24.3)	18.7 (20.7)
	B	24.0 (26.8)	14.9 (17.7)	12.0 (14.3)		B	17.1 (20.1)	22.0 (24.3)	18.7 (20.7)
	C	20.1 (23.3)	14.9 (17.7)	16.8 (18.9)		C	17.1 (20.1)	22.0 (24.3)	17.8 (18.8)
	D	20.1 (23.3)	14.9 (17.7)	12.0 (14.2)		D	17.1 (20.1)	22.0 (24.3)	18.7 (20.9)

Table S12. DFT Electronic Energies (Δ*E*, in kcal·mol-1) and Gibbs Energies (in brackets) ((Δ*G*), in kcal·mol-1) of the MEPs for (*S*)-MeG Insertion promoted by (*R*)-3B. Preferred Paths are Reported in Bold.

Site	Path	Wrapping mode of **TS1-TS2**	**TS1** Δ*E* (Δ*G*)	**TSα** Δ*E* (Δ*G*)	**TS2** Δ*E* (Δ*G*)
**B at B-chain**	M1-A	*fm*1-*fm*1 (*re*)	-1.3 (14.5)		2.7 (17.8)
	**M2-A**	***fm*1-*fm*2 (*si*)**	**0.9 (16.3)**		**1.3 (15.8)**
	M3-A	*fm*1-*ff* (*si*)	0.9 (16.3)	2.7 (17.5)	4.1 (18.2)
**A at B-chain**	**M1-A**	***fm*1-*fm*1 (*si*)**	**2.3 (16.6)**		**3.0 (18.8)**
	M2-B	*fm*2-*fm*1 (*si*)	3.0 (20.1)		3.0 (18.8)
	M3-A	*fm*1-*ff* (*re*)	5.2 (21.6)	1.0 (17.7)	-0.6 (14.3)

Table S13. TSs Gibbs energies (Δ*G*, with respect to (*R*)-3A + monomer in kcal/mol) for all reaction paths computed for the (*S*)-MeG insertion into the Al-A-chain bond at site B. Values calculated in toluene (dichloromethane, DCM, reported in brackets). In bold red and green the low-lying paths in toluene and DCM, respectively.

Monomer	*S*				Monomer	*S*			
Site	B				Site	B			
Chain	A				Chain	A			
Enantioface	*re*				Enantioface	*si*			
		**TS1**		**TS2**			**TS1**		**TS2**
Mechanism 1 (**M1**)	A	11.8 (14.7)		17.1 (19.2)	Mechanism 1 (**M1**)	A	12.1 (15.2)		18.9 (22.0)
	B	15.4 (17.2)		17.8 (19.4)		B	13.8 (16.7)		15.0 (18.1)
	C	**14.0** **(16.5)**		**13.0** **(15.7)**		C	17.6 (20.1)		16.9 (19.9)
		**TS1**		**TS2**			**TS1**		**TS2**
Mechanism 2 (**M2**)	A	11.8 (14.7)		17.8 (19.4)	Mechanism 2 (**M2**)	A	12.1 (15.2)		15.0 (18.1)
	B	15.4 (17.2)		17.1 (19.2)		B	13.8 (16.7)		18.9 (22.0)
		**TS1**	**TSα**	**TS2**			**TS1**	**TSα**	**TS2**
Mechanism 3 (**M3**)	A	11.8 (14.7)	15.1 (17.7)	13.0 (15.7)	Mechanism 3 (**M3**)	A	12.1 (15.2)	17.6 (20.3)	16.9 (19.9)
	B	15.4 (17.2)	15.1 (17.7)	13.0 (15.7)		B	13.8 (16.7)	17.6 (20.3)	16.9 (19.9)
	C	14.0 (16.5)	15.1 (17.7)	17.1 (19.2)		C	17.6 (20.1)	17.6 (20.3)	18.9 (22.0)
	D	14.0 (16.5)	15.1 (17.7)	17.8 (19.4)		D	17.6 (20.1)	17.6 (20.3)	15.0 (18.1)

Table S14. TSs Gibbs energies (ΔG, with respect to (*R*)-3A + monomer in kcal/mol) for all reaction paths computed for the (*S*)-MeG insertion into the Al-A-chain bond at site A. Values calculated in toluene (dichloromethane, DCM, reported in brackets). In bold red and green the low-lying paths in toluene and DCM, respectively.

Monomer	*S*				Monomer	*S*			
Site	A				Site	A			
Chain	A				Chain	A			
Enantioface	*re*				Enantioface	*si*			
		**TS1**		**TS2**			**TS1**		**TS2**
Mechanism 1 (**M1**)	A	16.2 (19.5)		15.3 (16.9)	Mechanism 1 (**M1**)	A	12.7 (15.1)		20.0 (22.4)
	B	18.7 (20.9)		13.0 (15.4)		B	11.9 (15.2)		20.1 (23.3)
	C	**14.4** **(16.8)**		**11.2** **(13.8)**		C	n.c.		16.8 (19.5)
		**TS1**		**TS2**			**TS1**		**TS2**
Mechanism 2 (**M2**)	A	16.2 (19.5)		13.0 (15.4)	Mechanism 2 (**M2**)	A	12.7 (15.1)		20.1 (23.3)
	B	18.7 (20.9)		15.3 (16.9)		B	11.9 (15.2)		20.0 (22.4)
		**TS1**	**TSα**	**TS2**			**TS1**	**TSα**	**TS2**
Mechanism 3 (**M3**)	A	16.2 (19.5)	14.7 (17.3)	11.2 (13.8)	Mechanism 3 (**M3**)	A	12.7 (15.1)	20.1 (22.8)	16.8 (19.5)
	B	18.7 (20.9)	14.7 (17.3)	11.2 (13.8)		B	11.9 (15.2)	20.1 (22.8)	16.8 (19.5)
	C	14.4 (16.8)	14.7 (17.3)	15.3 (16.9)		C	n.c.	20.1 (22.8)	20.0 (22.4)
	D	14.4 (16.8)	14.7 (17.3)	13.0 (15.4)		D	n.c.	20.1 (22.8)	20.1 (23.3)

Table S15. **TSs Gibbs energies (Δ*G*, with respect to (*R*)-3B + monomer in kcal/mol) for all reaction paths computed for the (*R*)-MeG insertion into the Al-B-chain bond at site B. Values calculated in toluene (dichloromethane, DCM, reported in brackets). In bold red and green the low-lying paths in toluene and DCM, respectively.**

Monomer	*R*				Monomer	*R*			
Site	B				Site	B			
Chain	B				Chain	B			
Enantioface	*re*				Enantioface	*si*			
		**TS1**		**TS2**			**TS1**		**TS2**
Mechanism 1 (**M1**)	A	12.8 (16.4)		15.5 (18.2)	Mechanism 1 (**M1**)	A	14.2 (16.8)		18.6 (21.5)
	B	21.7 (24.9)		15.0 (16.7)		B	14.5 (17.6)		19.1 (22.8)
	C	18.0 (20.8)		12.4 (15.6)		C	17.2 (20.0)		13.0 (15.7)
		**TS1**		**TS2**			**TS1**		**TS2**
Mechanism 2 (**M2**)	A	**12.8** **(16.4)**		**15.0** **(16.7)**	Mechanism 2 (**M2**)	A	14.2 (16.8)		19.1 (22.8)
	B	21.7 (24.9)		15.5 (18.2)		B	14.5 (17.6)		18.6 (21.5)
		**TS1**	**TSα**	**TS2**			**TS1**	**TSα**	**TS2**
Mechanism 3 (**M3**)	A	12.8 (16.4)	15.4 (18.6)	12.4 (15.6)	Mechanism 3 (**M3**)	A	14.2 (16.8)	18.6 (21.3)	13.0 (15.7)
	B	21.7 (24.9)	15.4 (18.6)	12.4 (15.6)		B	14.5 (17.6)	18.6 (21.3)	13.0 (15.7)
	C	18.0 (20.8)	15.4 (18.6)	15.5 (18.2)		C	17.2 (20.0)	18.6 (21.3)	18.6 (21.5)
	D	18.0 (20.8)	15.4 (18.6)	15.0 (16.7)		D	17.2 (20.0)	18.6 (21.3)	19.1 (22.8)

Table S16. TSs Gibbs energies (Δ*G*, with respect to (*R*)-3B + monomer in kcal/mol) for all reaction paths computed for the (*R*)-MeG insertion into the Al-B-chain bond at site A. Values calculated in toluene (dichloromethane, DCM, reported in brackets). In bold red and green the low-lying paths in toluene and DCM, respectively.

Monomer	*R*				Monomer	*R*			
Site	A				Site	A			
Chain	B				Chain	B			
Enantioface	*re*				Enantioface	*si*			
		**TS1**		**TS2**			**TS1**		**TS2**
Mechanism 1 (**M1**)	A	11.0 (15.0)		n. c.	Mechanism 1 (**M1**)	A	20.1 (23.0)		13.3 (16.1)
	B	21.8 (22.9)		15.9 (17.9)		B	18.0 (21.2)		12.4 (16.3)
	C	18.0 (20.9)		19.5 (22.9)		C	18.7 (21.9)		12.7 (15.5)
		**TS1**		**TS2**			**TS1**		**TS2**
Mechanism 2 (**M2**)	A	**11.0** **(15.0)**		**15.9** **(17.9)**	Mechanism 2 (**M2**)	A	20.1 (23.0)		12.4 (16.3)
	B	21.8 (22.9)		n. c.		B	18.0 (21.2)		13.3 (16.1)
		**TS1**	**TSα**	**TS2**			**TS1**	**TSα**	**TS2**
Mechanism 3 (**M3**)	A	11.0 (15.0)	14.8 (18.4)	19.5 (22.9)	Mechanism 3 (**M3**)	A	20.1 (23.0)	14.6 (17.8)	12.7 (15.5)
	B	21.8 (22.9)	14.8 (18.4)	19.5 (22.9)		B	18.0 (21.2)	14.6 (17.8)	12.7 (15.5)
	C	18.0 (20.9)	14.8 (18.4)	n. c.		C	18.7 (21.9)	14.6 (17.8)	13.3 (16.1)
	D	18.0 (20.9)	14.8 (18.4)	15.9 (17.9)		D	18.7 (21.9)	14.6 (17.8)	12.4 (16.3)

Table S17. DFT Electronic Energies (Δ*E*, in kcal·mol^{-1}) and Gibbs Energies (in brackets) ((Δ*G*), in kcal·mol^{-1}) of the MEPs for (*R*)-MeG Insertion promoted by (*R*)-3B. Preferred Paths are Reported in Bold.

Site	Path	Wrapping mode of **TS1-TS2**	**TS1** Δ*E* (Δ*G*)	**TSα** Δ*E* (Δ*G*)	**TS2** Δ*E* (Δ*G*)
**B at B-chain**	M1-A	*fm*1-*fm*1 (*re*)	1.4 (16.4)		3.8 (18.2)
	**M2-A**	***fm*1-*fm*2 (*re*)**	**1.4 (16.4)**		**2.0 (16.7)**
	M3-A	*fm*1-*ff* (*re*)	1.4 (16.4)	3.4 (18.6)	0.9 (15.6)
**A at B-chain**	M1-B	*fm*2-*fm*2 (*si*)	3.3 (21.2)		1.0 (15.3)
	**M2-A**	***fm*1-*fm*2 (*re*)**	**0.6 (15.0)**		**3.8 (17.9)**
	M3-D	*ff*-*fm*2 (*re*)	6.3 (20.9)	1.8 (18.4)	3.8 (17.9)

Table S18. Electronic Energies (ΔΔE) and Gibbs Energies (ΔΔG, in brackets) in kcal·mol^{-1} for the regioselectivity of (*S*)-MeG and (*R*)-MeG ROP promoted by (*R*)-3B depending on the computational approach.

Monomer	Preferred site	SP methodology		
		B3LYP-D3(BJ) ΔΔE (ΔΔG)	M06 ΔΔE (ΔΔG)	ωB97XD ΔΔE (ΔΔG)
**(*S*)-MeG**	B + B-chain	1.7 (2.5)	1.5 (2.2)	3.2 (4.0)
**(*R*)-MeG**	B + B-chain	1.8 (1.2)	1.6 (1.0)	1.2 (0.7)

Table S19. TSs Gibbs energies (ΔG, with respect to (R)-3A + monomer in kcal/mol) for all reaction paths computed for the (R)-MeG insertion into the Al-A-chain bond at site A. Values calculated in toluene (dichloromethane, DCM, reported in brackets). In bold red and green the low-lying paths in toluene and DCM, respectively.

Monomer	*R*				Monomer	*R*			
Site	A				Site	A			
Chain	A				Chain	A			
Enantioface	*re*				Enantioface	*si*			
		**TS1**		**TS2**			**TS1**		**TS2**
Mechanism 1 (**M1**)	A	10.8 (13.7)		25.3 (28.3)	Mechanism 1 (**M1**)	A	16.1 (18.4)		16.5 (19.3)
	B	15.1 (17.3)		13.7 (16.0)		B	15.0 (17.5)		12.8 (14.4)
	C	n. c.		12.7 (15.3)		C	15.0 (17.5)		13.1 (15.1)
		**TS1**		**TS2**			**TS1**		**TS2**
Mechanism 2 (**M2**)	A	**10.8** **(13.7)**		**13.7** **(16.0)**	Mechanism 2 (**M2**)	A	16.1 (18.4)		12.8 (14.4)
	B	15.1 (17.3)		25.3 (28.3)		B	15.0 (17.5)		16.5 (19.3)
		**TS1**	**TSα**	**TS2**			**TS1**	**TSα**	**TS2**
Mechanism 3 (**M3**)	A	10.8 (13.7)	15.3 (18.1)	12.7 (15.3)	Mechanism 3 (**M3**)	A	16.1 (18.4)	17.3 (19.8)	13.1 (15.1)
	B	15.1 (17.3)	15.3 (18.1)	12.7 (15.3)		B	15.0 (17.5)	17.3 (19.8)	13.1 (15.1)
	C	n. c.	15.3 (18.1)	25.3 (28.3)		C	15.0 (17.5)	17.3 (19.8)	16.5 (19.3)
	D	n. c.	15.3 (18.1)	13.7 (16.0)		D	15.0 (17.5)	17.3 (19.8)	12.8 (14.4)

Table S20. TSs Gibbs energies (Δ*G*, with respect to (*R*)-3A + monomer in kcal/mol) for all reaction paths computed for the (*R*)-MeG insertion into the Al-A-chain bond at site B. Values calculated in toluene (dichloromethane, DCM, reported in brackets). In bold red and green the low-lying paths in toluene and DCM, respectively.

Monomer	*R*				Monomer	*R*			
Site	B				Site	B			
Chain	A				Chain	A			
Enantioface	*re*				Enantioface	*si*			
		**TS1**		**TS2**			**TS1**		**TS2**
Mechanism 1 (**M1**)	A	12.3 (14.6)		18.9 (21.5)	Mechanism 1 (**M1**)	A	12.9 (15.0)		18.3 (21.2)
	B	13.4 (15.5)		15.3 (17.4)		B	11.5 (13.8)		19.9 (22.6)
	C	n.c.		15.6 (18.2)		C	**14.0** **(16.1)**		**14.5** **(16.7)**
		**TS1**		**TS2**			**TS1**		**TS2**
Mechanism 2 (**M2**)	A	12.3 (14.6)		15.3 (17.4)	Mechanism 2 (**M2**)	A	12.9 (15.0)		19.9 (22.6)
	B	13.4 (15.5)		18.9 (21.5)		B	11.5 (15.5)		18.3 (21.2)
		**TS1**	**TSα**	**TS2**			**TS1**	**TSα**	**TS2**
Mechanism 3 (**M3**)	A	12.3 (14.6)	15.6 (18.5)	15.6 (18.2)	Mechanism 3 (**M3**)	A	12.9 (15.0)		14.5 (16.7)
	B	13.4 (15.5)	15.6 (18.5)	15.6 (18.2)		B	11.5 (15.5)		14.5 (16.7)
	C	n.c.	15.6 (18.5)	18.9 (21.5)		C	14.0 (16.1)		18.3 (21.2)
	D	n.c.	15.6 (18.5)	15.3 (17.4)		D	14.0 (16.1)		19.9 (22.6)

References

(1) M. J. Frisch, G. W. T., H. B. Schlegel, G. E. Scuseria, M. A. Robb, J. R. Cheeseman, G. Scalmani, V. Barone, G. A. Petersson, H. Nakatsuji, X. Li, M. Caricato, A. Marenich, J. Bloino, B. G. Janesko, R. Gomperts, B. Mennucci, H. P. Hratchian, J. V. Ortiz, A. F. Izmaylov, J. L. Sonnenberg, D. Williams-Young, F. Ding, F. Lipparini, F. Egidi, J. Goings, B. Peng, A. Petrone, T. Henderson, D. Ranasinghe, V. G. Zakrzewski, J. Gao, N. Rega, G. Zheng, W. Liang, M. Hada, M. Ehara, K. Toyota, R. Fukuda, J. Hasegawa, M. Ishida, T. Nakajima, Y. Honda, O. Kitao, H. Nakai, T. Vreven, K. Throssell, J. A. Montgomery, Jr., J. E. Peralta, F. Ogliaro, M. Bearpark, J. J. Heyd, E. Brothers, K. N. Kudin, V. N. Staroverov, T. Keith, R. Kobayashi, J. Normand, K. Raghavachari, A. Rendell, J. C. Burant, S. S. Iyengar, J. Tomasi, M. Cossi, J. M. Millam, M. Klene, C. Adamo, R. Cammi, J. W. Ochterski, R. L. Martin, K. Morokuma, O. Farkas, J. B. Foresman, and D. J. Fox *Gaussian 09 Rev. E01*, Wallingford, CT, 2016.

(2) Becke, A. D., Density-functional thermochemistry. III. The role of exact exchange. *J. Chem. Phys.* **1993,** *98*, 5648-5652.

(3) Lee, C.; Yang, W.; Parr, R. G. Development of the Colle-Salvetti correlation-energy formula into a functional of the electron density. *Phys. Rev. B* **1988,** *37*, 785-789.

(4) McLean, A. D.; Chandler, G. S. Contracted Gaussian basis sets for molecular calculations. I. Second row atoms, Z=11–18. *J. Chem. Phys.* **1980,** *72*, 5639-5648.

(5) Binkley, S.; Pople, J. A.; Hehre, W. J. Self-Consistent Molecular Orbital Methods. 21. Small Split-Valence Basis Sets for First-Row Elements. *J. Am. Chem. Soc.* **1980,** *102*, 939-947.

(6) Schäfer, A.; Huber, C.; Ahlrichs, R. Fully optimized contracted Gaussian basis sets of triple zeta valence quality for atoms Li to Kr. *J. Chem. Phys.* **1994,** *100*, 5829-5835.

(7) Grimme, S.; Antony, J.; Ehrlich, S.; Krieg, H. A consistent and accurate ab initio parametrization of density functional dispersion correction (DFT-D) for the 94 elements H-Pu. *J. Chem. Phys.* **2010,** *132*, 154104.

(8) Barone, V.; Cossi, M. Quantum Calculation of Molecular Energies and Energy Gradients in Solution by a Conductor Solvent Model. *J. Phys. Chem. A* **1998,** *102*, 1995-2001.

(9) Cossi, M.; Rega, N.; Scalmani, G.; Barone, V. Energies, structures, and electronic properties of molecules in solution with the C-PCM solvation model. **2003,** *24*, 669-681.

(10) D'Alterio, M. C.; De Rosa, C.; Talarico, G. Stereoselective Lactide Polymerization: The Challenge of Chiral Catalyst Recognition. *ACS Cat.* **2020,** *10*, 2221-2225.

(11) D'Alterio, M. C.; De Rosa, C.; Talarico, G., Syndiotactic PLA from *meso*-LA polymerization at the Al-chiral complex: a probe of DFT mechanistic insights. *Chem. Commun.* **2021,** *57*, 1611-1614.

(12) Falivene, L.; Cavallo, L.; Talarico, G., Buried Volume Analysis for Propene Polymerization Catalysis Promoted by Group 4 Metals: A Tool for Molecular Mass Prediction. *ACS Catal.* **2015,** *5*, 6815-6822.

(13) Falivene, L.; Barone, V.; Talarico, G., Unraveling the role of entropy in tuning unimolecular *vs.* bimolecular reaction rates: The case of olefin polymerization catalyzed by transition metals. *Mol. Catal.* **2018,** *452*, 138-144.

(14) Lu, Y.; Coates, G. W., Pairing-Enhanced Regioselectivity: Synthesis of Alternating Poly(lactic-co-glycolic acid) from Racemic Methyl-Glycolide. *J. Am. Chem. Soc.* **2023,** *145* (41), 22425-22432.

(15) Zhao, Y.; Truhlar, D. G., The M06 suite of density functionals for main group thermochemistry, thermochemical kinetics, noncovalent interactions, excited states, and transition elements: two new functionals and systematic testing of four M06-class functionals and 12 other functionals. *Theor. Chem. Acc.* **2008,** *120*, 215-241.

(16) Chai, J.-D.; Head-Gordon, M., Long-range corrected hybrid density functionals with damped atom–atom dispersion corrections. *Phys. Chem. Chem. Phys.* **2008,** *10*, 6615-6620.
